# MCM-BP Is Required for Repression of Life-Cycle Specific Genes Transcribed by RNA Polymerase I in the Mammalian Infectious Form of *Trypanosoma brucei*

**DOI:** 10.1371/journal.pone.0057001

**Published:** 2013-02-25

**Authors:** Hee-Sook Kim, Sung Hee Park, Arthur Günzl, George A. M. Cross

**Affiliations:** 1 Laboratory of Molecular Parasitology, The Rockefeller University, New York, New York, United States of America; 2 Department of Genetics and Developmental Biology, University of Connecticut Health Center, Connecticut, United States of America; 3 Department of Molecular, Microbial and Structural Biology, University of Connecticut Health Center, Connecticut, United States of America; University of Nottingham, United Kingdom

## Abstract

*Trypanosoma brucei* variant surface glycoprotein (*VSG*) expression is a classic example of allelic exclusion. While the genome of *T. brucei* contains >2,000 *VSG* genes and *VSG* pseudogenes, only one allele is expressed at the surface of each infectious trypanosome and the others are repressed. Along with recombinatorial *VSG* switching, allelic exclusion provides a major host evasion mechanism for trypanosomes, a phenomenon known as antigenic variation. To extend our understanding of how trypanosomes escape host immunity by differential expression of *VSG*s, we attempted to identify genes that contribute to *VSG* silencing, by performing a loss-of-silencing screen in *T. brucei* using a transposon-mediated random insertional mutagenesis. One identified gene, which we initially named *LOS1*, encodes a *T. brucei* MCM-Binding Protein (TbMCM-BP). Here we show that TbMCM-BP is essential for viability of infectious bloodstream-form (BF) trypanosome and is required for proper cell-cycle progression. Tandem affinity purification of TbMCM-BP followed by mass spectrometry identified four subunits (MCM4-MCM7) of the *T. brucei* MCM complex, a replicative helicase, and MCM8, a subunit that is uniquely co-purified with TbMCM-BP. TbMCM-BP is required not only for repression of subtelomeric *VSG*s but also for silencing of life-cycle specific, insect-stage genes, procyclin and procyclin-associated genes (*PAG*s), that are normally repressed in BF trypanosomes and are transcribed by RNA polymerase I. Our study uncovers a functional link between chromosome maintenance and RNA pol I-mediated gene silencing in *T. brucei*.

## Introduction

*Trypanosoma brucei* is a protozoan parasite that causes African sleeping sickness in humans and a similar disease in livestock, in sub-Saharan Africa. *T. brucei* cycles between the insect vector (tsetse) and mammalian hosts. Two proliferating life-cycle forms, the insect-midgut procyclic form (PF) and the mammalian bloodstream form (BF), can be cultured and genetically manipulated. A single species of *VSG* is expressed at any time in each BF parasite, where VSG homogenously coats the parasite surface. All *VSG*s are repressed in PF, whose surface is coated with members of a small family of proteins called procyclins.

Four distinct loci contain *VSG*s and *VSG* peudogenes [1]. The majority of *VSG*s reside in minichromosomes and telomere-distal (sometimes called ‘chromosome-internal’) arrays, but they appear to lack promoters. *VSG*s in BF are transcribed by RNA polymerase I (Pol I) from about 15 subtelomeric polycistronic ‘Bloodstream-form *VSG* Expression Sites’ (BES) [2], [3]. These *VSG*s are located adjacent to telomere repeats and ∼50 kb downstream of their promoters. Only one BES is transcriptionally active and expresses one *VSG* in each bloodstream form cell whereas the remaining *VSG*s are repressed [1]. In the infectious metacyclic (tsetse) stage, *VSGs* are transcribed from monocistronic subtelomeric expression sites that are distinct from BESs [4], [5].

Silencing of BES promoters and *VSG*s appears to be regulated by factors involved in telomere dynamics and chromatin modification. Loss of TbSIR2-rp1 (a *T. brucei* SIR2 homologue) or TbRAP1 (a telomere-binding protein) led to derepression of silent *VSGs* [6], [7]. Several studies have demonstrated that chromatin remodeling and histone marks may be involved in BES promoter and/or *VSG* silencing; TbISWI, a member of the ISWI family of chromatin remodeling complexes [8]; TbDOT1B, a histone methyltransferase [9]; TbHAT1, a histone acetyltransferase [10]; TbDAC3, a histone deacetylase [11]; and TbSPT16, a subunit of the chromatin remodeling FACT complex [12]. Derepression of silent *VSG*s was also observed in BF cells depleted of chromatin assembly factors, TbASF1A and TbCAF-1b, and a linker histone, H1 [13], [14].

Thus far, studies of *T. brucei* antigenic variation have been largely relied on searching for homologues that have been characterized in other organisms. However, because *T. brucei* proteins are highly divergent from those of other model organisms, due to early separation during evolution, homologue searches have limitations. To better understand the molecular mechanisms underlying *VSG* silencing and to isolate components relevant to *VSG* gene silencing, we performed a large-scale forward genetic screen in *T. brucei*: a ‘loss-of-silencing’ (LOS) screen. We isolated 19 *los* clones that had impaired ability to repress a silent BES. One of these genes, *LOS1*, encodes a *T. brucei* MCM-Binding Protein (MCM-BP). MCM-BP is a component of a replication complex that consists of subunits of the Mini-Chromosome Maintenance (MCM) complex and of MCM-BP [15]–[17].

DNA replication initiates with binding of the Origin Recognition Complex (ORC) to replication origins and recruitment of members of the pre-replication complex (pre-RC), including CDC6, CDT1, CDC45/GINS complex and MCM complex. This activates the replication origins and proceeds to replication by DNA polymerases (reviewed in [18]). Along with these core replication proteins, several of alternative replication complexes have been identified and extensively studied in yeasts and human. Alternative replication complexes structurally resemble replication complexes and participate in the maintenance of chromosome integrity without being directly involved in DNA replication. They are either composed of specific subunits or deviate from canonical complexes by one or more subunits. For example, the 9-1-1 complex (RAD9-HUS1-RAD1), a donut-shaped heterotrimer, resembles the homotrimeric Prolierating Cell Nuclear Antigen (PCNA, a replication clamp), a replication factor that is required for the processivity of DNA polymerase [19]. PCNA loading onto DNA requires the Replication Factor C (RFC) complex, the clamp loader, which is composed of five subunits RFC1-5 [20]. RAD24 or CTF18 replaces RFC1 and forms a RAD24-RFC2-5 or CTF18-RFC2-5, and these complexes are involved in DNA damage checkpoint and chromosome segregation [21], [22]. The MCM-BP complex is the most recent addition to the list of alternative replication complexes. The MCM complex, a replication helicase, consists of six subunits, MCM2-MCM7 (reviewed in [23]). In plants, the MCM-BP, also known as the ETG1 (E2F target gene 1), interacts with all MCM subunits, MCM2-7. The ETG1 is required for DNA replication, repair, and sister-chromatid cohesion [17], [24]. In human and fission yeast, MCM-BP replaces MCM2 and forms a complex with MCM3-7 [15], [16]. An *in vitro* study with *Xenopus* egg extracts showed that MCM-BP is required for removal of the MCM complex from DNA at the end of the S-phase to ensure replication licensing for the next round [25].

DNA replication proteins have been implicated in gene silencing. Subunits of ORC, a hexameric complex, participate in heterochromatin-mediated silencing in several organisms by interacting with the heterochromatin proteins SIR2-4 and HP1 [26]–[29]. Subunits of the MCM complex are also involved in gene expression through interactions with histone H3, transcription factor STAT1, and RNA Pol II in human cells [30]–[33]. In the budding yeast temperature-sensitive mutant *mcm5*, subtelomeric chromatin was more loosely packed and subtelomeric genes were up-regulated at the non-permissive temperature [34]. In addition, the temperature sensitivity of *mcm5* was suppressed by overexpressing *TRA1*, a component of the SAGA chromatin-remodeling complex [34].

In *T. brucei*, we are beginning to understand mechanism of DNA replication with recent identification of several subunits of ORC, components of pre-RC and MCM complex [35]–[37]. Recently, replication origins have been mapped in all 11 megabase chromosomes using chromatin immunoprecipitation (chIP) of TbORC1 [38] and BrdU labeling demonstrated that TbORC1 is required for *de novo* nuclear DNA synthesis both in BF and PF trypanosomes [39]. The majority of replication origins appear to overlap with start sites of polycistronic transcription units in *T. brucei* [38]. TbORC1 binding sites are also enriched along BESs [38] and at telomeres [39]. Similar to ORC1 in other model organisms, TbORC1 seems to have functions outside of DNA replication. Depletion of TbORC1 increased expression levels of BES-associated silent *VSG*s both in BF and PF trypanosomes [39], and of metacyclic *VSG*s in PF cells [38]. TbORC1 is also required for *VSG* switching, particularly a mechanism involving a transcriptional switch between the active and a silent BES [39].

In this study, we performed a forward genetic screen to isolate genes that are responsible for maintaining the repressed status of a silent BES, using a *mariner*-transposon random insertional mutagenesis, which led to the isolation of an allele of *TbMCM-BP*. TbMCM-BP is essential for cell viability in the infectious BF stage. Deficiency of TbMCM-BP is associated with derepression of silent *VSG*s and silent procyclic genes that are transcribed by RNA Pol I, and with a novel cell-cycle defect. Tandem affinity purification of TbMCM-BP demonstrated that TbMCM-BP is strongly associated with four *T. brucei* MCM-subunits, MCM4-MCM7, and MCM8, a subunit that is uniquely co-purified with MCM-BP only in *T. brucei*. It is notable that it is the first time that a *VSG* silencing factor has been selected by a phenotype-based large-scale screening approach and that *T. brucei* is the first organism in which MCM-BP was identified in such way.

## Results

### Loss-of-silencing screen and isolation of *Trypanosoma brucei* MCM-BP

We performed a large-scale random insertional mutagenesis screen to isolate genes that participate in the mechanism of *VSG* silencing. The screen was carried out in PF *T. brucei*, for several reasons. First, the screen requires a single-copy autonomously replicating plasmid, which is not available in BF trypanosomes. Secondly, BF trypanosomes occasionally switch their *VSG*s at a frequency of ∼1–2×10^−5^ [40], [41]. Therefore, switchers can be selected as false-positive clones. Thirdly, for a large-scale screen to work in a diploid organism (a haploid meiotic stage seems to occur only inside the insect vector [42]), it is essential to generate a sufficiently large pool of mutants with potential homozygous gene disruptions. PF cells can grow to a 10–20-fold higher density than BF cells. Finally, all *VSG*s, including all BES-associated *VSG*s, are silent in PF *T. brucei*. Given all these factors, PF offered a greater potential for the success of an initial screen. Once genes are isolated, they can be characterized for their roles in BF trypanosomes, where antigenic variation is physiologically relevant.

**Figure 1:**
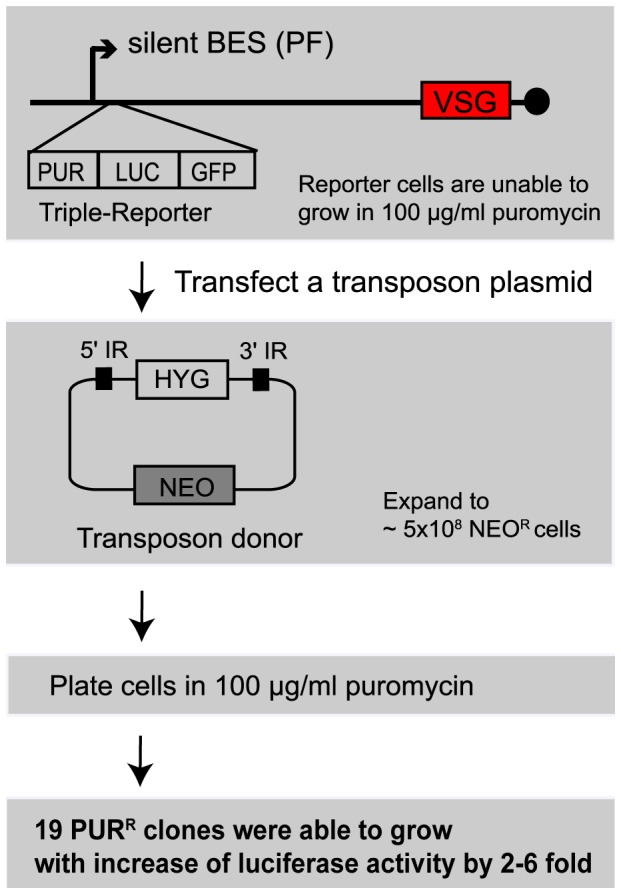
Loss-of-silencing (LOS) screen. A triple reporter cassette carrying puromycin-resistance (*PUR*), luciferase (*LUC*), and emerald GFP (em*GFP*) genes was targeted adjacent to a silent BES promoter in procyclic form *T. brucei* and selected with 1 µg/ml puromycin. The reporter cells are unable to grow at a higher concentration of puromycin (100 µg/ml), due to BES promoter silencing. After stably integrating a transposase at an array of tubulin genes, cells were mutagenized using a transposon insertion. The transposon plasmid carries a *mariner*-donor containing *HYG* marker flanked by inverted repeats (IRs). Expansion of neomycin-selected cells was distributed in 96-well plates with 100 µg/ml of puromycin. Only transposon-mutant cells that had lost the ability to repress this silent BES locus can grow at this concentration of puromycin but parental cells cannot. 19 *PUR^R^* clones were obtained and increased luciferase activity by 2–6-fold (Table S1). Black circle is telomere.

To generate a reporter cell line for a loss-of-silencing (LOS) screen, BES11 (containing *VSG*16) was targeted with a triple-reporter cassette containing puromycin-resistance (*PUR*), luciferase (*LUC*), and emerald-GFP (em*GFP*) genes, adjacent to its promoter (Figure 1). Reporter cells were selected in 1 µg/ml puromycin, as a low level of transcription occurs close to a ‘silent’ BES promoter [43], allowing drug selection at a low concentration. A *mariner* transposase gene was integrated into the tubulin locus and insertional mutagenesis was induced by transfection of the donor-plasmid pSGL35 [44] containing a hygromycin-resistance gene (*HYG*) flanked by *mariner* inverted repeats (IRs) and a neomycin-resistance gene (*NEO*) for the selection of cells transformed by the plasmid. Neomycin-resistant cells (*NEO^R^*) were expanded to a population of ∼5×10^8^ cells, prior to selection with hygromycin. Cells can only become resistant to hygromycin once the donor cassette is transposed into a transcribed chromosomal orientation.

Transpositions that led to increased expression of *PUR* marker (‘*los*’ clones) were isolated by simultaneously selecting with 40 µg/ml of hygromycin and 100 µg/ml of puromycin. Parental cells cannot grow at this concentration of puromycin while *los* mutants that lost the ability to repress expression of *PUR* gene should. 19 *PUR^R^* clones were obtained (Table S1). Luciferase expression was also increased in all clones (2–6-fold increase compared to the parental line). Transposon target sites were mapped by inverse PCR and sequencing. We were unable to identify target site(s) in three *PUR^R^* clones. In some clones, the transposon-*HYG* donor was integrated at multiple locations, for example clones 2 and 12. In six cases, transposons were inserted at intergenic regions, which may disrupt functions of untranslated regions (UTRs) and affect expression levels of neighboring genes.

Five clones had an identical insertion in the middle of one allele of the *Tb927.7.1770* (gene ID number in http://www.genedb.org and http://tritrypdb.org) open reading frame (ORF), so were not independent events, causing an about 3–6-fold increase in luciferase activity near the reporter-tagged silent BES promoter (Table S1, clone numbers are highlighted in blue). However, unlike what we expected, only one allele of *Tb927.7.1770* was disrupted in all five clones. We provisionally named the disrupted gene *LOS1* (loss-of-silencing gene 1).

**Figure 2:**
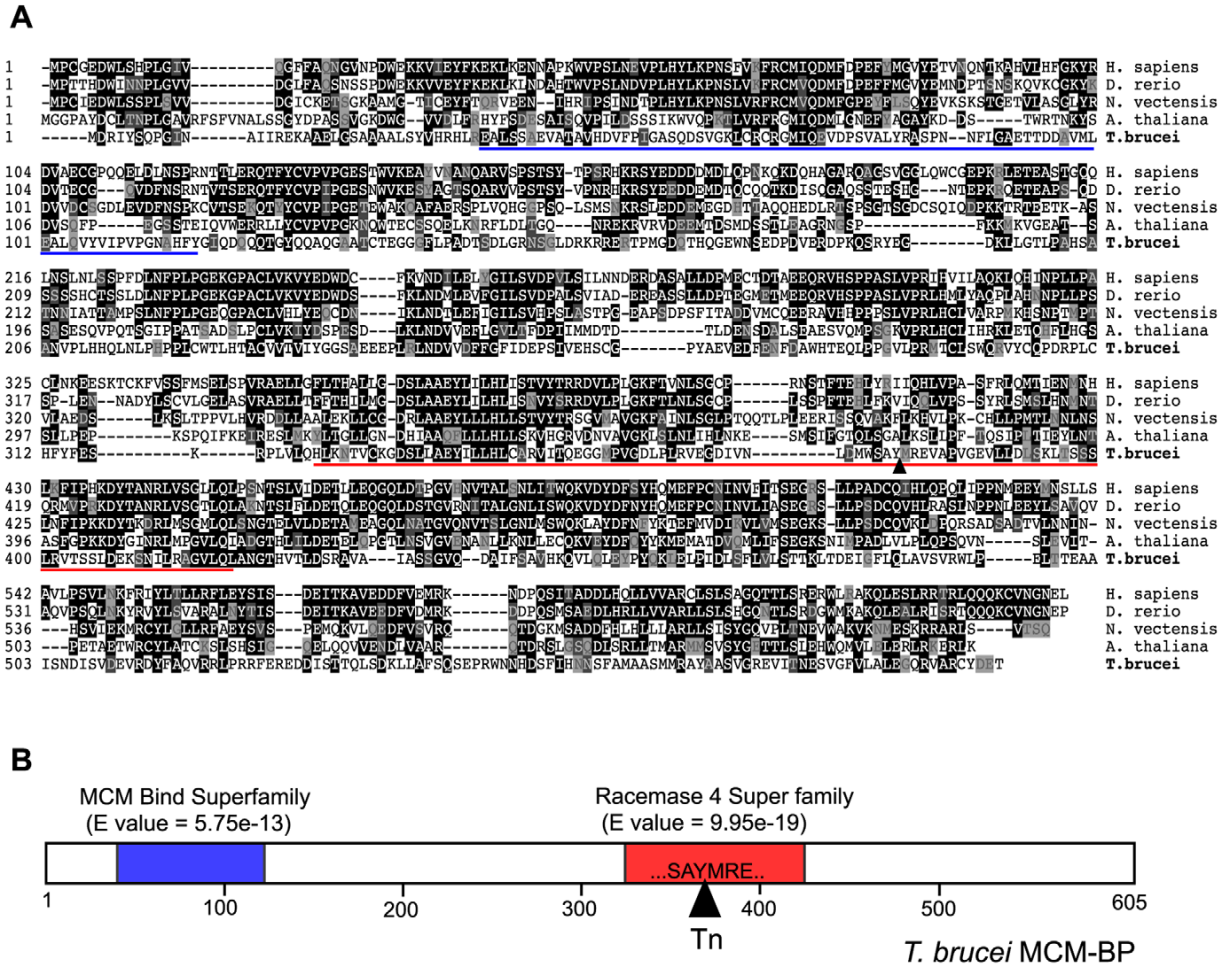
LOS1 shows sequence similarities to the MCM-Binding Protein (MCM-BP). (A) Alignment of LOS1 with human, fish, worm and plant MCM-BP is shown. BLAST searches identify that all of MCM-BP including LOS1 contain two families of sequences, called ‘MCM Bind Superfamily’ (underlined in blue) and ‘Racemase 4 Super family’ (underlined in red). (B) A schematic diagram of *T. brucei* MCM-BP. A black triangle in the ‘Racemase 4 Superfamily’ domain indicates the site where a *mariner* transposon (Tn) was inserted.

BLAST searches with LOS1 identified sequence similarities to the MCM-Binding Protein (MCM-BP) that interacts with subunits of the MCM complex [15]–[17]. Figure 2A shows a sequence alignment of LOS1 with human, fish, worm, and plant MCM-BP, all of which contain two domains, ‘MCM Bind Superfamily’ and ‘Racemase 4 Super family’. LOS1 appears to have these domains and the transposon was inserted in the middle of the ‘Racemase 4 Superfamily’ region (Figure 2B). A functional study in fission yeast with a series of truncated MCM-BP proteins showed that the N-terminal half of MCM-BP was sufficient for the interaction with MCM4, but was unable to rescue the lethality of an *mcm-BP* null mutant [16]. Therefore, it is possible that the transposon-targeted *los1* allele may express an N-terminal portion of TbMCM-BP, which may act as a dominant negative allele in *los1* heterozygous clones, resulting in the loss of BES promoter silencing. LOS1 will be referred to as TbMCM-BP henceforth.

### TbMCM-BP is essential for viability of BF *T. brucei*

To investigate whether TbMCM-BP is required for *VSG* silencing in BF, where antigenic variation occurs, we attempted to make a homozygous *TbMCM-BP* deletion mutant in BF but we were not able to delete both alleles, suggesting that *TbMCM-BP* is an essential gene. To deplete the *TbMCM-BP* transcript by RNAi, the wild-type *TbMCM-BP* allele in heterozygous *TbMCM-BP^−/+^* was first epitope-tagged *in situ* with myc. The *TbMCM-BP^−/MYC^* cells did not show any growth defect, verifying that the tagged version is functional. The *TbMCM-BP^−/MYC^* strain was then transfected with a tetracycline-inducible construct for *TbMCM-BP* dsRNA expression [45]. Two RNAi clonal cell lines showed growth defects upon tetracycline addition that correlated with the disappearance of TbMCM-BP-myc protein (Figure S1).

**Figure 3:**
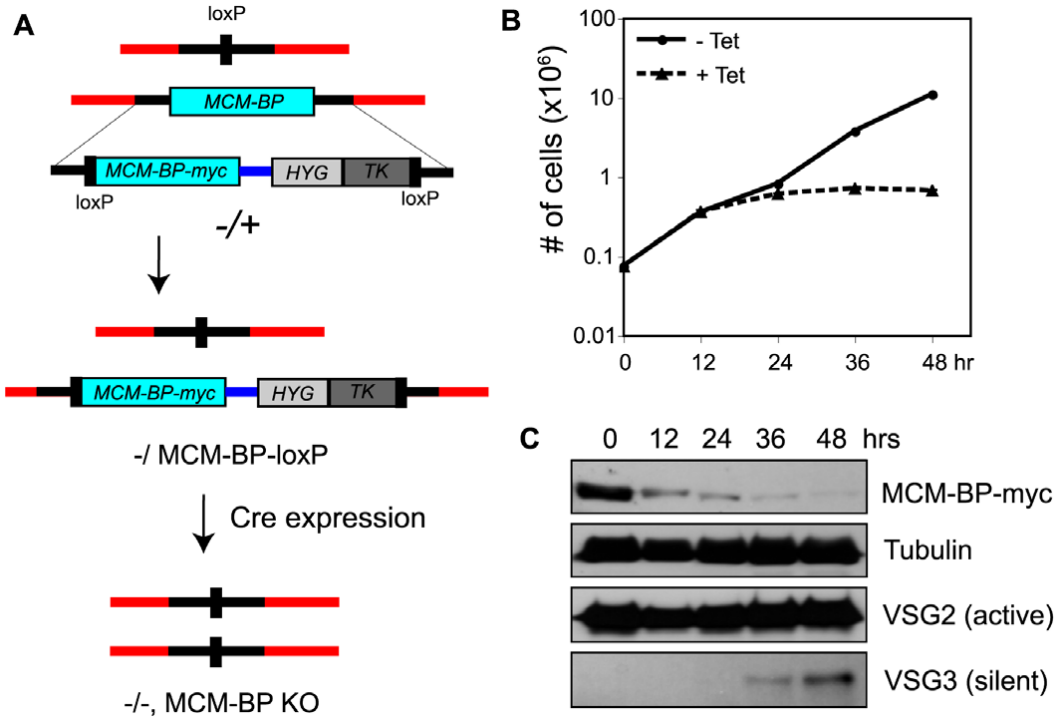
TbMCM-BP is essential for viability of BF *T. brucei*. (A) *TbMCM-BP* conditional knock out (cKO) strategy using Cre-loxP. In a *TbMCM-BP* single KO strain, remaining wild type *TbMCM-BP* allele was replaced with *MCM-BP-myc-HYG-TK* cassette flanked by loxPs. Homozygously deleted *TbMCM-BP* double KO mutation can be obtained by expressing Cre-recombinase, which was stably inserted at an rDNA locus. (B) *T. brucei* MCM-BP is essential for cell viability of the infectious BF *T. brucei*. Cell growth was arrested upon *TbMCM-BP* removal by Cre expression. (C) Depletion of TbMCM-BP protein upon Cre expression by immunoblot. Tubulin, VSG2, and VSG3 were analyzed by immunoblot. VSG3 protein was detected in TbMCM-BP deficient cells.

RNAi knockdown may have off-target effects and is sometimes not very efficient. Although TbMCM-BP depletion correlated with cell growth defect, depletion was not efficient. It was incomplete in RNAi line 2, and in cell line 1, silencing of *TbMCM-BP* gene took 96 hr (about 16 generations) and cells seemed to escape from RNAi silencing after 144 hr. To have a definite answer for the essentiality of *TbMCM-BP* in BF *T. brucei*, and to generate a more reliable cell line to examine cellular functions of TbMCM-BP, we constructed a conditional-knock-out (cKO) cell line (Figure 3A). One allele of *TbMCM-BP* was deleted using a dual positive and negative selectable *PUR-TK* gene fusion flanked with loxP sites. Cre-recombinase under the control of tetracycline was stably introduced to remove the *PUR-TK* marker. In this *TbMCM-BP^−/+^* heterozygous strain, an endogenous *TbMCM-BP* allele was then replaced with a cassette that contains *MCM-BP-myc* followed by *HYG-TK*. Two loxP sites flank the cassette so that the *MCM-BP-myc* and *HYG-TK* can be removed by conditionally expressing Cre-recombinase (Figure 3A). *TbMCM-BP* cKO cells were harvested at 12-hr intervals for 48 hr upon Cre induction. Cell growth was arrested 12 hr after Cre induction and TbMCM-BP-myc gradually disappeared (Figure 3B and 3C), confirming that *TbMCM-BP* is an essential gene and demonstrating that the conditional KO system is much more reliable than RNAi-mediated gene knockdown which showed no cell growth arrest even after 144 hr of RNAi induction.

### TbMCM-BP is required for *VSG* silencing in BF *T. brucei*

**Figure 4:**
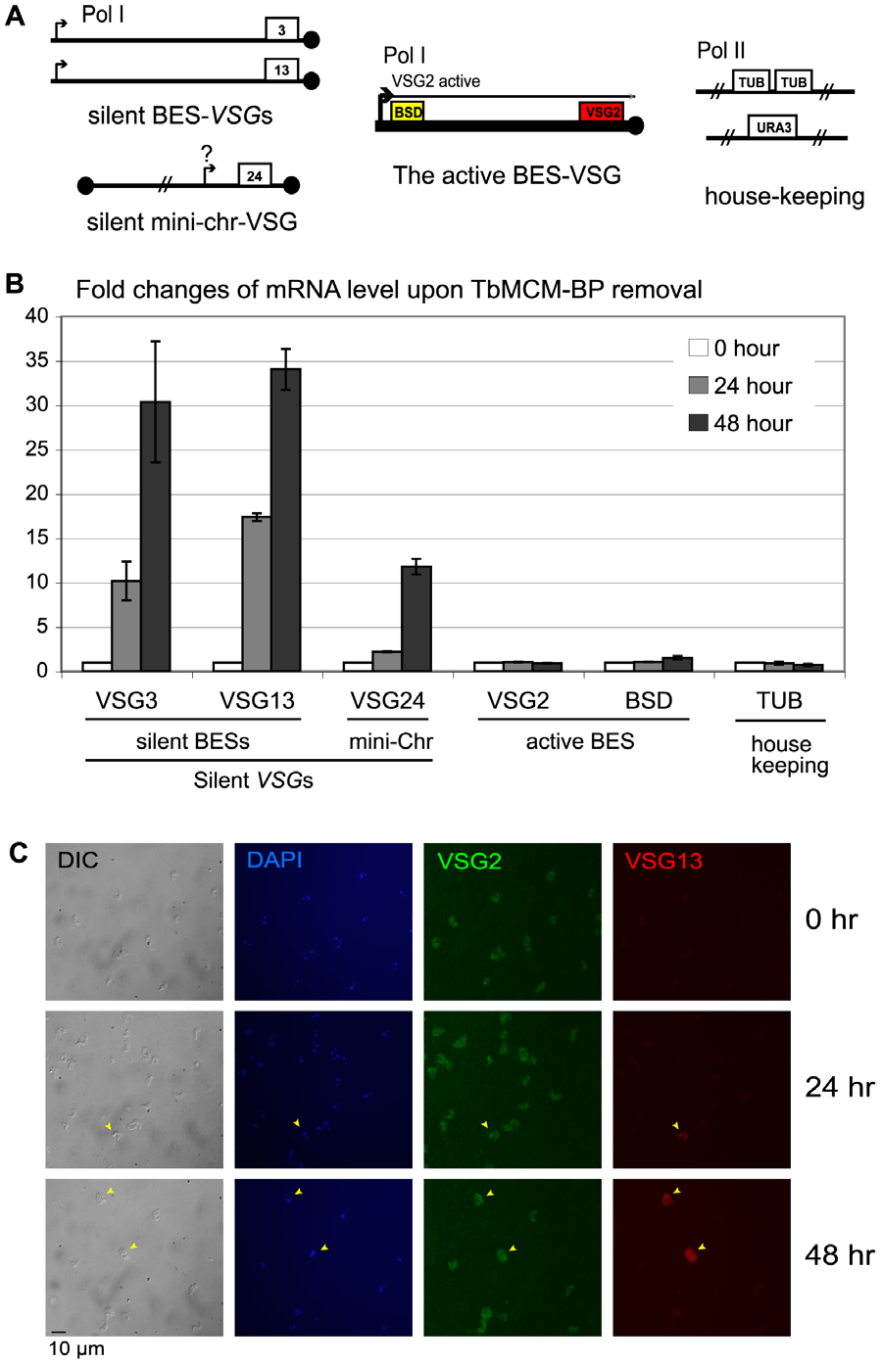
TbMCM-BP is required for *VSG* silencing in BF *T. brucei*. (A) Diagrams of genes and their chromosomal locations: silent BES-linked *VSG*s (*VSG*3 and *VSG*13), a minichromosomal *VSG*24, the active *VSG*2 and *BSD* marker, and two housekeeping control genes (*TUB* and *URA3*). Black circles are telomere repeats. (B) TbMCM-BP deficiency derepresses silent *VSG* expression. After 24 and 48 hr of post-Cre-induction, mRNA levels of each genes described in (A) were measured and normalized to *TbURA3* mRNA level. Fold changes relative to Cre-uninduced cells (0 hr) are plotted. Standard deviations are shown as error bars. (C) Expression of silent VSG13 protein in the VSG2 active BF cells. Cells were fixed with 1% paraformaldehyde and permeabilized with 0.2% NP40. Samples were incubated with chicken anti-VSG2 and rabbit anti-VSG13, and then incubated with secondary antibodies (green for VSG2 and red for VSG13). DNA was stained with DAPI.

To examine whether *VSG* silencing was lost in TbMCM-BP-depleted BF trypanosomes, we assessed the expression levels of several *VSG* genes in different chromosomal locations by reverse transcription quantitative PCR (RT-qPCR). We quantified derepression of three silent *VSG*s: two silent BES-linked subtelomeric *VSG*s, *VSG*3 (also called *VSG*224) and *VSG*13 (*VSG*113), and a minichromosomal *VSG*, *VSG*24 [46] (Figure 4A). We also measured expression of *BSD* and *VSG*2 from the active BES1 and of two chromosome-internal housekeeping control genes (*TbTUB* and *TbURA3*). Cre-expression was induced to knock out *TbMCM-BP-myc* and cells were harvested 24 and 48 hr after Cre induction. Levels of mRNA were quantified by RT-qPCR and values were normalized to the level of *TbURA3* mRNA. Figure 4B shows fold changes relative to the *TbMCM-BP* wild type cells (0 hr). Expression of silent BES-linked *VSG*3 and *VSG*13 increased 10- and 18-fold after 24 hr and 30- and 35-fold after 48 hr upon *TbMCM-BP* removal. Interestingly, expression of minichromosomal *VSG* (*VSG*24) increased 2- and 12-fold after 24 and 48 hrs after *TbMCM-BP* removal. *T. brucei* contains a large number of small chromosomes called minichromosomes (MCs), which are 30–150 kb in size. According to genome-wide high-throughput sequencing analysis, it appears that there are ∼60 MC *VSG*s in the genome of this strain of *T. brucei* (GAMC, unpublished data). Mapping of 17 MCs showed a core structure consisting of a large central palindromic 177-bp repeat region and telomeric ends, some of which contain *VSG*s [47]. These subtelomeric *VSG*s do not seem to have their own Pol I promoter [47]. Depletion of TbMCM-BP increased expression levels of BES-associated subtelomeric *VSG*s and a minichromosomal subtelomeric *VSG*, but did not significantly change the expression level of the active *VSG*2 and *BSD*, or of another chromosome-internal housekeeping gene, *TbTUB*. Our data suggest that TbMCM-BP may have roles in silencing subtelomeric *VSG*s.

We observed expression of silent VSG3 by immunoblotting after *TbMCM-BP* removal (Figure 3C, bottom), which demonstrated that silent *VSG* transcripts can be translated. We were able to detect VSG3 because it migrates slower than VSG2, eliminating the possibility that the antibody cross-reacted with the major VSG2 band. However, we were unable to detect changes in VSG13 protein expression, as the VSG13 band overlaps with that of VSG2. To ensure that elevated levels of silent *VSG* expression are due to loss of silencing and not due to *VSG* switching, we visualized the active and silent VSGs by immunofluorescence. Cells were double-stained with anti-VSG2 (active, green) and anti-VSG3 (silent, red) or VSG13 (silent, red) antibodies. We observed cells co-expressing the active VSG2 and the silent VSG3 (data not shown) and cells co-expressing the active VSG2 and the silent VSG13 (Figure 4C, yellow arrowheads) upon *TbMCM-BP* removal. However, silent VSGs were detectable by IF only in a subset of cells, probably because the levels of these partially derepressed VSGs were generally very low. We did not detect any switched cells that no longer expressed VSG2. Collectively, out data confirm that TbMCM-BP is required to maintain the repressed status of silent *VSG*s.

### TbMCM-BP is required for full repression of silent RNA Pol I promoters in BF *T. brucei*

The loss-of-silencing phenotype of *TbMCM-BP* mutation was identified originally in a PF cell line with reporter genes inserted immediately downstream of a silent BES promoter. Silencing near BES promoters appears to be mechanistically distinct from silencing near telomeres (reviewed in [48]).

**Figure 5:**
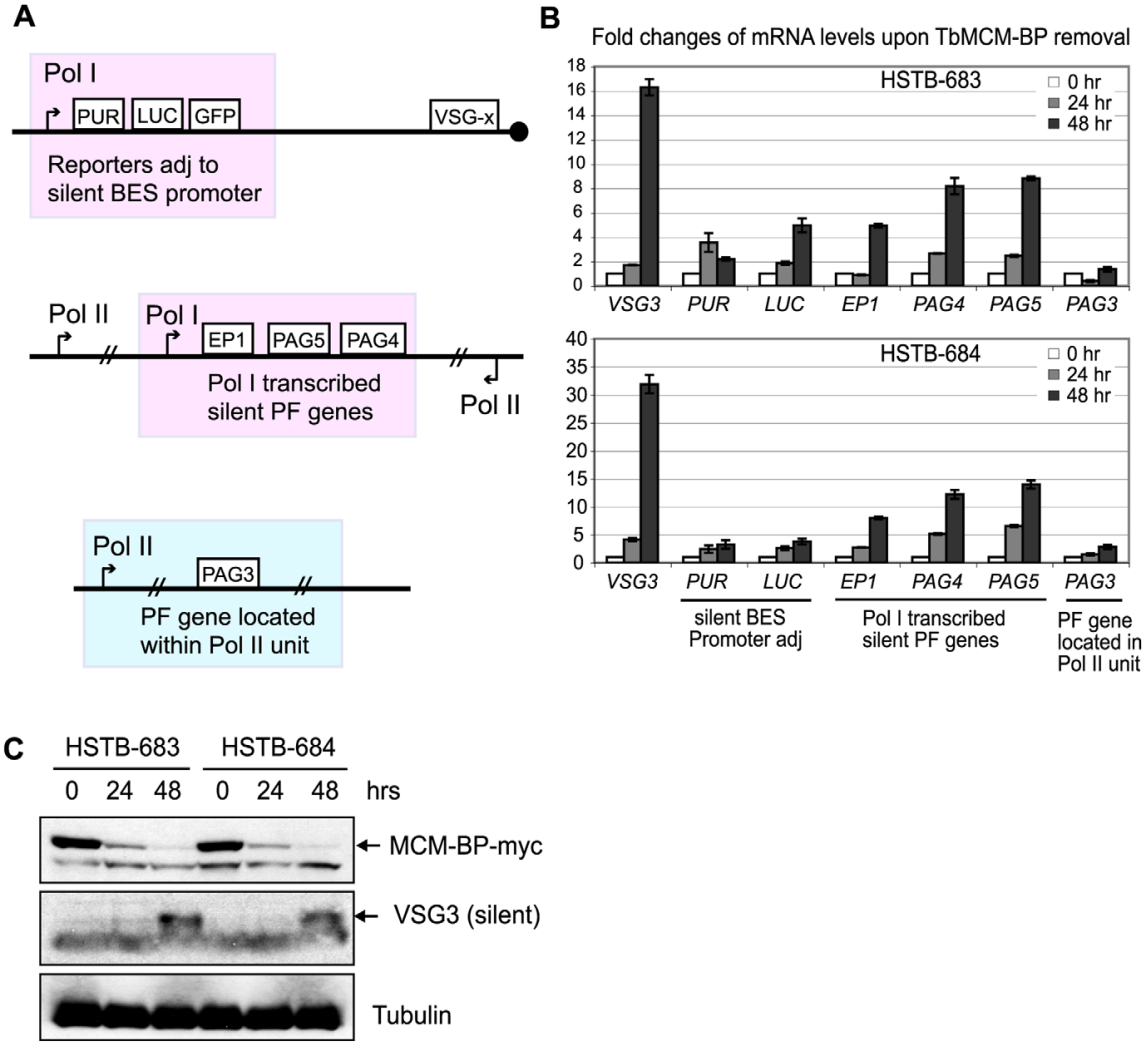
TbMCM-BP is required for full repression of silent RNA Pol I promoters in BF *T. brucei*. (A) Diagrams of genes and their locations: a triple reporter in a silent BES, procyclic genes under a RNA Pol I promoter, and a procyclic gene within a RNA Pol II transcription unit. (B) TbMCM-BP deficiency increased expression of silent genes transcribed from RNA Pol I promoter. After 24 and 48 hr of post-Cre-induction, mRNA levels of each genes described in (A) were measured and normalized to *TbURA3* mRNA level. Fold changes relative to Cre-uninduced cells (0 hr) are plotted. Standard deviations are shown as error bars. (C) Depletion of TbMCM-BP protein upon Cre induction by immunoblot. VSG3 expression was used as a positive control for RT-qPCR and immunoblot.

To examine whether TbMCM-BP also influences the silencing at a BES promoter in BF *T. brucei*, we inserted the same *PUR-LUC-GFP* triple reporter cassette at a silent BES promoter in the TbMCM-BP-conditional KO cell line and obtained two independently targeted cell lines (HSTB-683 and -684). Expression levels of *PUR* and *LUC* genes (top diagram in Figure 5A) were quantified by RT-qPCR and values were normalized to the level of *TbURA3* mRNA. *VSG3* derepression was used as a positive control in RT-qPCR and immunoblots (Figure 5B and 5C). TbMCM-BP depletion caused only minor effects on BES promoter silencing with maximum increases of about 4-fold in *PUR* and of about 5-fold in Luciferase gene expression (Figure 5B). We also observed increased expression of emGFP by flow cytometry analysis (data not shown). The derepression was not as dramatic as at the silent *VSG*s, probably because TbMCM-BP is required largely for subtelomeric silencing within the BES units or because, as is known [49], [50], repression is already weaker near silent promoters than closer to the telomeres. Figure 5C shows TbMCM-BP depletion and VSG3 expression after Cre induction by immunoblot analysis. Tubulin was used as a loading control.

Insect-midgut PF trypanosomes express several procyclic-specific genes, called procyclin (*GPEET* and *EP* variants) and procyclin-associated genes (*PAG*s) that are also transcribed from an RNA Pol I promoter, which is less active in BF than in PF [51]. Some *PAG*s are located downstream of procyclin genes under the RNA Pol I promoter (diagram in Figure 5A, middle) and some, for example *PAG3* (Figure 5A, bottom), are located within RNA Pol II polycistronic units. To examine whether TbMCM-BP plays specific roles in RNA Pol I-mediated transcription, we measured expression levels of *EP1* and *PAG*s in the absence of TbMCM-BP. As shown in Figure 5B, depletion of TbMCM-BP derepressed *EP1*, *PAG5* and *PAG4*, which are transcribed by RNA Pol I, but did not significantly affect *PAG3* expression directed by RNA Pol II. These results collectively indicate that TbMCM-BP is required for full repression of BES promoters and of RNA Pol I-transcribed, PF-specific genes in BF trypanosomes.

### Accumulation of G2 and anucleated cells (zoids) in *TbMCM-BP* null mutant

**Figure 6:**
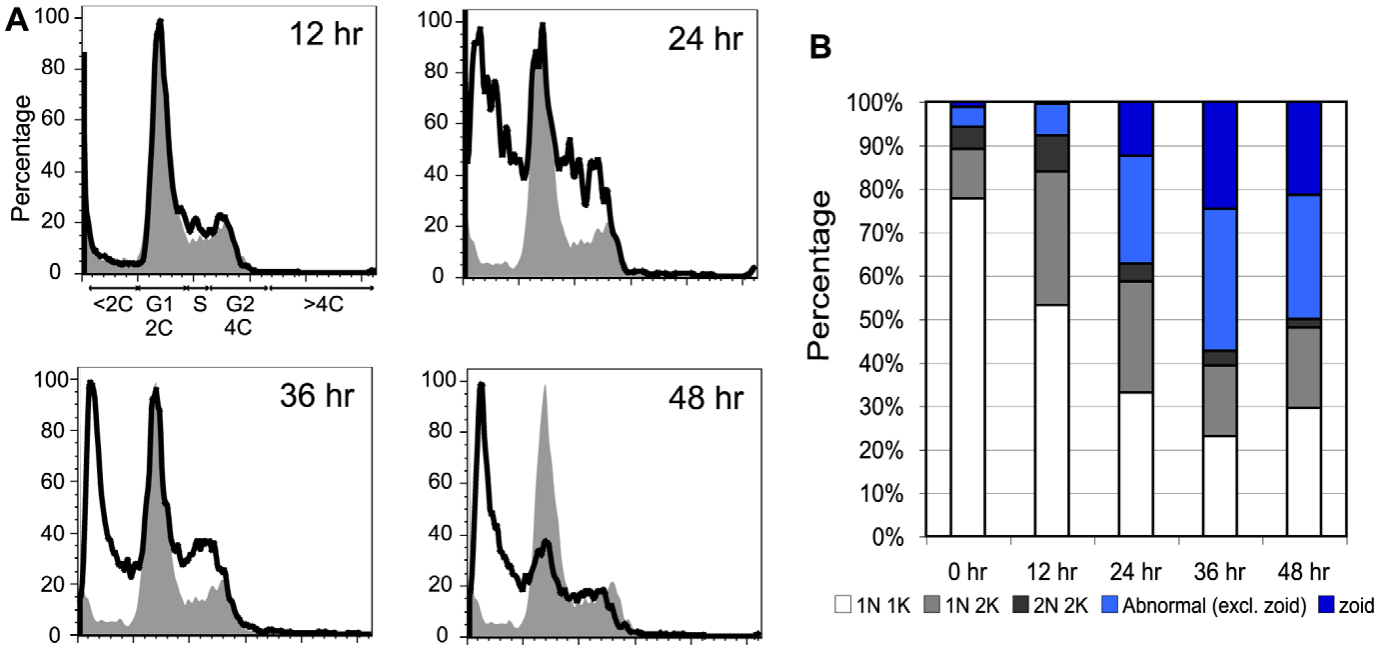
Accumulation of G2 and zoid cells in *TbMCM-BP*-deficient BF *T. brucei*. (A) The accumulation of G2 and <2C cells upon *TbMCM-BP* removal by flow cytometry. (B) The accumulation of the 1N2K (G2) and zoid cells upon *TbMCM-BP* removal by DAPI staining.

MCM-BP deficiency is associated with cell-cycle arrest in G2/M and activates the G2 checkpoint in plant, human and fission yeast [16], [17], [24], [52]. In addition, MCM-BP is required for replication termination, by removing MCM at the end of S-phase in *Xenopus* [25]. To examine whether TbMCM-BP deficiency impairs cell-cycle progression in BF trypanosomes, we monitored cell-cycle progression after Cre-induced *TbMCM-BP* removal by flow cytometry, staining bulk DNA with propidum iodide. As shown in Figure 6A, control cells (grey area, 0 hr) showed normal cell cycle profiles: 2C (G1 phase, 56.5%), 4C (G2 phase, 14.9%), and a population between 2C and 4C (S phase, 8.67%). Twelve hours after Cre induction, the cell-cycle profile remained normal, but peaks representing S and G2 became higher at 24 and 36 hrs, and lower after 48 hr, indicating that TbMCM-BP-depleted cells arrest at S-G2. Interestingly, <2C content cells gradually increased over time. The accumulation and then subsequent reduction of the S-G2 population and the continuing increase of <2C cells suggest that depletion of TbMCM-BP induces a G2 arrest, and that chromosomes segregate before S-phase completes, and, therefore, that TbMCM-BP may coordinate cell cycle arrest and cytokinesis.

In *T. brucei*, replication and segregation of kinetoplast DNA (K) precede those of nuclear DNA (N) [53], so cells at different stages can be distinguished by their NK configurations. 1N1K content indicates that cells are either in G1 or S phase. When cells are entering into late S/G2, they show 1N2K content with two closely spaced kinetoplasts. Mitotic cells have 2N2K content, with two Ks are already separated but 2N remain close to each other. Post-mitotic cells have 2N2K with separated daughter nuclei and kinetoplasts. Representatives of cells with these DNA contents are shown in Figure S2B (0 hr). To further confirm the cell cycle defects observed from the flow cytometry, we examined TbMCM-BP cKO cells stained with DAPI after Cre induction (Figure 6B). Before Cre induction (0 hr), the majority of control cells were 1N1K (78%, white bars), and G2 and M/post-M cells were 11% (light grey) and 5% (dark grey), respectively. After 12 hr of Cre induction, the G2 population increased from 11% to 31%. At 24, 36, and 48 hrs post-induction, G2 cells accumulated further.

Interestingly, the number of abnormal cells greatly increased over time, reaching up to 58% 36 hr post-induction (Figure 6B, blue bars). The most prominent abnormality caused by TbMCM-BP deficiency is accumulation of anucleated cells (0N0K, 0N1K, 0N2K), also known as zoids [54], [55]. These are probably the majority of >2C cells observed in the flow cytometry analysis. As zoids started accumulating at 24 hr post Cre induction, following 1N2K (G2 or late S) accumulation at 12 hr (Figure 6B), some of G2-arrested mother cells may have segregated into a zoid and 4C progeny. Other abnormal cells were observed less frequently (Figure S2), for example, cells that appeared to have less than 1N and a fragmented nucleus, probably due to defective DNA replication. Other cells showed defective chromosome segregation, exhibiting an elongated nucleus, DNA bridging, absence of kinetoplast, and uncoupled segregation of N and K (for example cells with 2N content but 2K remained close to each other and cells with 1N content but 2 separated Ks). The cell cycle profiles of the conditional-KO mutant suggest that TbMCM-BP is required for proper cell-cycle progression, especially at S/G2 phase, and timely cell division.

PF and BF trypanosomes have different mitotic checkpoints. Deficiency of the mitotic-cyclin *CYC6* increased the appearance of zoids in PF, but caused the accumulation of multinucleated endoreduplicated cells in BF [56]. Furthermore, mutations in *T. brucei* cell cycle and mitotic checkpoint genes encoding Polo-like kinase 1, and cdc2-related kinases [56]–[60] as well as TbTRF (a telomere-binding protein) [61] and TbSPT16 [12] all increased multinucleated cells in BF. In addition, depletion of replication factors, TbORC1 or subunits of MCM complex was not associated with accumulation of zoid cells in BF [36], [37], [39]. The accumulation of zoids observed in TbMCM-BP deficient cells is therefore a novel phenotype that has not been observed in any mutation of BF trypanosomes examined.

### *T. brucei* MCM-BP co-purifies with MCM4-MCM8

**Figure 7:**
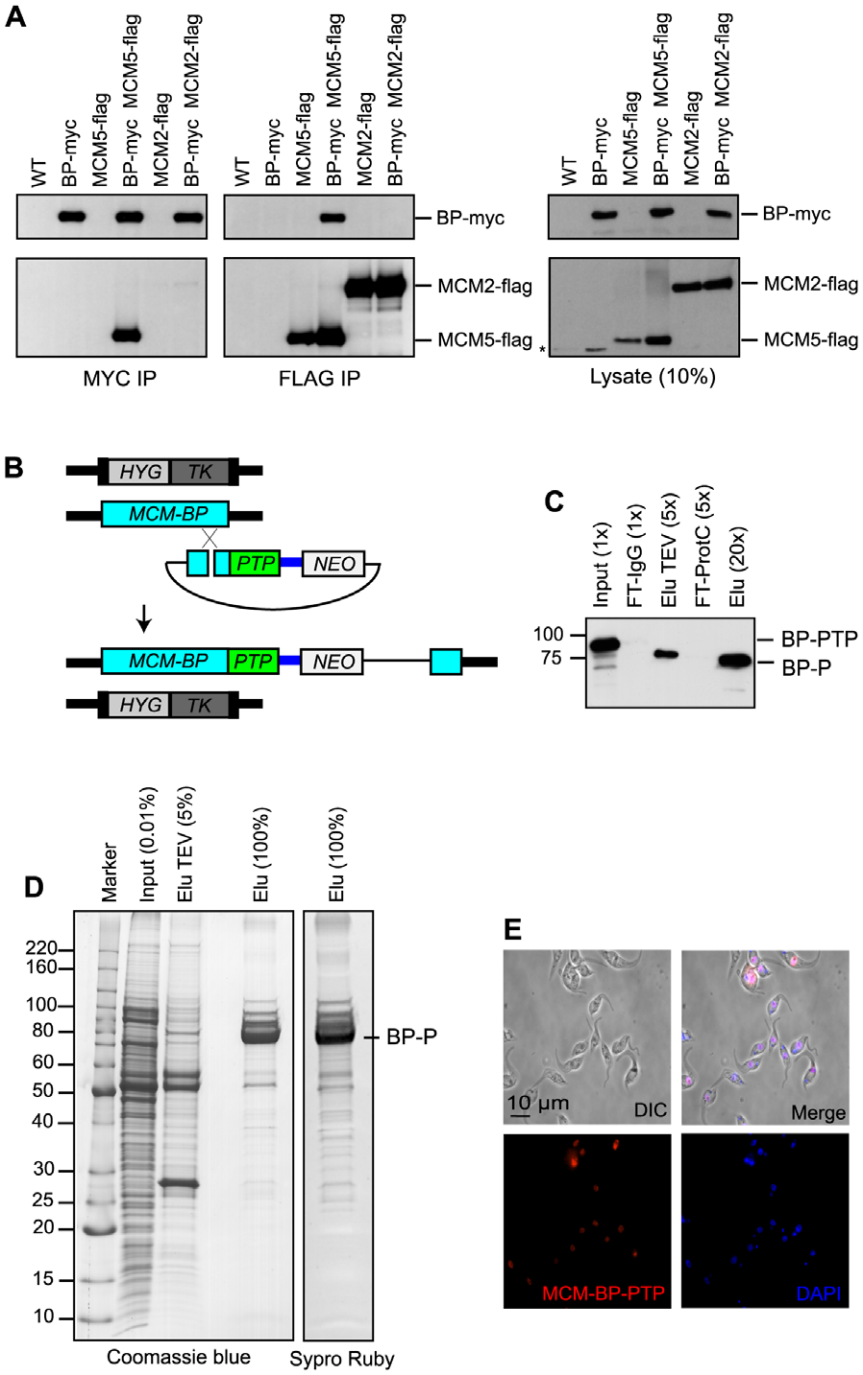
TbMCM-BP co-purifies with MCM4-MCM8. (A) TbMCM-BP interacts with MCM5 but not with MCM2 by co-immunoprecipitation. (B) Allele modification in cells exclusively expressing TbMCM-BP tagged with PTP. (C) Immunoblot monitoring of TbMCM-BP-PTP tandem affinity purification in extract (Input), IgG affinity chromatography flowthrough (FT-IgG), TEV protease eluate (Elu TEV), flowthrough of the anti-ProtC affinity chromatography (FT-ProtC), and final eluate (Elu). Note that TEV protease-mediated removal of the ProtA domains led to a size reduction of tagged TbMCM-BP (‘BP-PTP’ to ‘BP-P’). Relative amounts of aliquots are specified by x values. (D) Tandem affinity purification of TbMCM-BP. Final eluate and smaller aliquots of the TEV protease eluate and extract were separated on an SDS–gradient polyacrylamide gel and stained with Coomassie blue or Sypro Ruby. (E) TbMCM-BP is localized in the nucleus throughout the cell cycle in PF.

In human and yeast, MCM-BP replaces MCM2 and forms a complex with MCM3-7 [15], [62] while plant MCM-BP interacts with all MCM2-7 [17]. To characterize the *T. brucei* MCM-BP complex, we first examined interaction of TbMCM-BP with two MCM subunits, MCM2 (present in plant but not in human and yeast MCM-BP purification) and MCM5 (present in all MCM-BP complex purified so far). We carried out reciprocal co-immunoprecipitations (co-IPs) using epitope-tagged proteins expressed from their endogenous loci, TbMCM-BP-myc, MCM2-flag, or MCM5-flag in BF cells. The epitopes were inserted in heterozygous backgrounds (one allele deleted) to ensure functionalities of tagged versions. Lysates were immunoprecipitated and analyzed by immunoblotting using anti-myc or anti-flag antibodies. TbMCM-BP-myc pulled down only MCM5 but not MCM2 and TbMCM-BP-myc was consistently present only in MCM5-flag IP but not in MCM2-flag IP (Figure 7A). These results suggest that TbMCM-BP replaces MCM2 to form the *T. brucei* MCM-BP complex, similar to human and yeast MCM-BP.

To further characterize the TbMCM-BP complex, we isolated TbMCM-BP by tandem affinity purification (TAP) employing an approach that was developed in trypanosomes [63] and is based on the composite PTP tag consisting of a protein C epitope, a tobacco etch virus (TEV) protease site, and tandem protein A (ProtA) domains. As it is difficult to grow BF cells in a large quantity due to their sensitivity to cell density, TbMCM-BP was purified from PF trypanosomes, which grow to a 10–20-fold higher density.

We fused the PTP sequence to the 3′ end of the remaining *TbMCM-BP* allele in *TbMCM-BP^−/+^* PF cells by targeted insertion of plasmid pTbMCM-BP-PTP-NEO, thereby generating a cell line that expressed only C-terminally tagged TbMCM-BP (Figure 7B). Tandem affinity purification of tagged TbMCM-BP from crude extract prepared from this cell line was highly efficient as revealed by immunoblot monitoring of individual purification steps (Figure 7C). Staining of purified proteins after separation on an SDS–gradient polyacrylamide gel revealed a strong signal for TbMCM-BP as well as an array of bands that contained sub-stoichiometric amounts of co-purified proteins (Figure 7D). Most of these proteins appear to specifically interact with TbMCM-BP because the vast majority of these bands were not detectable in comparable purifications of other nuclear protein complexes in trypanosomes (for an example see [63]). Eight MCM proteins, MCM2-MCM9, were annotated (based on sequence homology) in the *T. brucei* genome. Liquid chromatography-tandem mass spectrometry analysis of the entire lane showed that TbMCM-BP co-purified with MCM4-MCM8, but not with MCM2, MCM3, and MCM9 (Table 1). MCM8 was not found in any MCM-BP complex that was isolated to date, which include complexes from human, plant, frog egg and fission yeast [15]–[17], [25]. The canonical *T. brucei* MCM complex has been recently characterized and consists of MCM2-7 [37]. Our protein interaction data suggest that the TbMCM-BP complex has an unusual composition. Furthermore, TbMCM-BP is located in the nucleus throughout the cell cycle in PF trypanosomes (Figure 7E).

**Table 1:** Mass spectrometric identification of *T. brucei* MCM subunits that co-purified with TbMCM-BP.

	Annotation^1^	Mol. mass^2^	# unique peptides^3^	Sequence coverage^4^	Peptide count^5^	Best ion score^6^	Protein score^7^
MCM-BP	Tb927.7.1770	68.2	27 .	49% .	404 .	133 .	8663 .
MCM4	Tb927.11.12250	93.3	31 .	47% .	70 .	102 .	888 .
MCM5	Tb927.11.5570	85.5	45 .	57% .	191 .	125 .	2093 .
MCM6	Tb927.11.11640	97.3	3 .	5% .	3 .	40 .	60 .
MCM7	Tb927.11.16140	82.1	42 .	44% .	124 .	87 .	713 .
MCM8	Tb927.10.10410	79.1	27 .	30% .	63 .	84 .	414 .

1 Accession number of the GeneDB/TriTrypDB databases.

2 Molecular mass in kDa.

3 Number of distinct peptide sequences identified for each protein.

4 Maximal sequence coverage of unique peptides identified from a single gel slice.

5 Total number of peptides identified for each co-purified protein.

6 Highest ion score among identified peptides for each protein (ion scores were considered significant at ≥22 with expectation values ≤0.05).

7 The protein score is the sum of highest ions score for each distinct sequence.

## Discussion

### Loss-of-silencing (LOS) screen using random mutagenesis in *T. brucei*

We performed our loss-of-silencing screen in PF, due to technical problems in BF and also due to some of advantages that the PF system offers, although monoallelic *VSG* expression is relevant in BF *T. brucei*. *T. brucei* is a diploid organism and transposon mutagenesis relies on subsequent second allele disruption by gene conversion (GC) from the first targeted allele to generate a pool of homozygous mutants, which is particularly critical for isolation of ‘loss-of-function’ alleles. It is therefore important that a screen should start with a sufficiently large pool of workable number of cells for the success of large-scale searches. Because *VSG*s are also repressed in PFs, which can grow to a 10–20 fold higher density than BF cells, PFs offered better chances for success of our initial screen.

Transposon insertional mutagenesis was induced by transfecting a transposon-donor plasmid [44] containing a *HYG* gene flanked by *mariner* inverted repeats (IRs) and *NEO* for the selection of cells transformed by the plasmid. *NEO*-resistant cells were expanded to a population of ∼5×10^8^ cells, prior to selection with hygromycin. Cells can only become resistant to hygromycin once the donor cassette is transposed into a transcribed chromosomal orientation. *HYG* transposition appears to be about 5% [44], suggesting that 2.5×10^7^ of 5×10^8^ *NEO*-resistant cells should be *HYG*-resistant, having transposon insertion somewhere in the genome. Previously, two independent homozygous glycosylation mutants were isolated from a large population of trypanosomes mutagenized with a *mariner*-transposon and the second allele seemed to have been disrupted by gene conversion by a *mariner*-targeted allele with an apparently significantly elevated GC rate, ∼2.5×10^−3^ [44]. This is about 250-fold higher than the GC rate measured at the *TbURA3* locus (∼1×10^−5^) [40]. If the elevated GC rate is a common feature of transposon-targeted loci, 6.25×10^4^ of 2.5×10^7^ *HYG*-resistant cells should be homozygous mutants, which is more than enough to cover the entire genome of *T. brucei* (∼8×10^3^ genes).

However, we were unable to identify any known genes that affect BES promoter silencing in PF, such as TbISWI, TbNLP, or TbDAC3 [8], [11], [64], [65]. In addition, only one allele was disrupted when we examined 8 transposon insertion sites in *los* clones (highlighted in yellow in Table S1), including the five *TbMCM-BP*-targeted *los1* clones. Therefore, the screen was not saturated and the previously observed high GC rate at the transposon-inserted site [44] was probably due to its position, rather than due to a universal effect of *mariner* transposition. If transposon-targeted regions have typical GC rates (∼1×10^−5^) [40], 2.5×10^7^ *NEO^R^ HYG^R^* cells should represent about 250 homozygous *los* mutants, covering only 3.1% of the genome.

One of the differences between the previous proof-of-principle transposon screen that isolated two homozygous glycosylation mutants and our LOS screen is that, in the former, a pool of transposon-mutagenized trypanosomes was strongly selected using eight sequential treatments with increasing amount of concanavalin A (conA, a drug that counter-selects glycosylation mutants), which may have allowed further enrichment of homozygous mutants. On the other hand, a trypanosome pool was treated one time with 100 µg/ml puromycin in our LOS screen, which may have limited the chance to accumulate homozygous mutants in the pool.

Despite the shortfall, it is noteworthy that our screen isolated a true positive clone that affects *VSG* silencing. An N-terminal half of fission yeast MCM-BP is sufficient for MCM4 interaction [16] and the transposon was targeted downstream of putative MCM binding domain in our *los1* clones. Therefore, it is possible that a C-terminally truncated Tbmcm-BP protein is expressed and acts as a dominant negative allele by competing with wild-type MCM-BP for the interaction with MCM subunits. In addition, in six clones, transposons were inserted at intergenic regions, which could disrupt functions of untranslated regions (UTRs) and affect expression levels of neighboring genes. In fact, in one *los* clone (clone 10), *Tb927.4.4520* expression was increased 5 fold proving that a transposon insertion can interfere with gene expression near its target site. Spontaneous loss of heterozygosity in *Leishmania major* has been reported to be 10^−4^∼10^−6^ and increased 20–1,000-fold after mutagen treatments [66]. Therefore, it may be possible to significantly increase the size of homozygous mutants in the pool to cover the entire genome of *T. brucei*, by sequentially treating transposon-targeted trypanosomes with increasing amount of puromycin together with mutagens. We conclude that our transposon-mediated random mutagenesis can be a useful tool for a large-scale genetic screen once properly tuned and it could isolate not only ‘loss-of-function’ mutants, but also ‘gain-of-function’ and overexpression mutants.

### MCM-BP in gene silencing in bloodstream form *T. brucei*

A number of recent studies demonstrated that chromosome maintenance factors are required for silencing of BES promoters and *VSG*s, and transcriptional *VSG* switching (*in situ* switching) in *T. brucei*. Depletion of TbORC1, a protein involved in replication initiation, derepressed BES-associated silent *VSG*s and metacyclic *VSG*s in BF trypanosomes and increased the *in situ* switching rate [35], [36], [38], [39]. Depletion of TbSCC1, a subunit of cohesin involved in chromosome segregation, also increased *in situ VSG* switching [67]. Derepression of silent *VSG*s was also observed in BF cells depleted of chromatin assembly factors, TbASF1A and TbCAF-1b, and the linker histone H1 [13], [14]. BF trypanosomes treated with aphidicolin, an inhibitor of DNA replication, increased expression of genes adjacent to a silent BES promoter in a dosage dependent manner but did not significantly change the expression level of silent *VSG*s [68]. At a high dose, aphidicolin induces global chromosome aberrations [69]. Interestingly, several of chromatin remodeling proteins showed similar phenotypes as an aphidicolin treatment. For example, TbISWI, TbSPT16, TbDAC3 and TbNLP showed strong effects near silent BES promoters without significantly affecting silencing near telomeres [8], [11], [12], [65]. Because we have limited mechanistic understanding of trypanosome DNA replication, chromatin assembly, and antigenic variation, it is not clear how these factors involved in BES and/or *VSG* silencing are linked. However, because TbMCM-BP-depleted cells showed stronger derepression near telomeres than silent BES promoters, TbMCM-BP should have roles in *VSG* silencing independently of chromatin remodeling proteins.

Recently, replication origins have been mapped along all 11 megabase chromosomes by TbORC1 chromatin IP [38]. Most replication origins appear to be located at the start of RNA pol II polycistronic transcription units. TbORC1 is also enriched along BES, including subtelomeric *VSG* regions [38], and TbORC1 associates with telomeres [39]. It is not known whether any of TbORC1 binding sites in BESs are utilized as replication origins but, given the high density of binding sites, it is likely that some of them might be required for cellular functions other than replication initiation. Human MCM-BP interacts with DBF4, a regulatory subunit of an early S-phase specific kinase complex DDK, which also contains CDC7 protein kinase [70]. Phosphorylation of MCM helicase by DDK is required for replication initiation, and MCM-BP partially inhibits phosphorylation of MCM by DDK [70] suggesting that MCM-BP has roles in replication initiation. In addition, overexpression of MCM-BP in fission yeast inhibited replication in S-phase and accumulated cells with less than 2C content and fragmented nuclei which could be due to a replication defect and/or to the uncoupling of completion of DNA replication and cell division [16]. On the other hand, MCM-BP is required specifically for replication termination by removing MCM helicase complex at the end of S-phase in *Xenopus* [25]. It is not clear whether TbMCM-BP is also required for replication initiation and termination. However, while silencing of *TbORC1*, *MCM* subunit genes, and *TbMCM-BP* affected G2 progression in BF cells, only TbMCM-BP depletion caused significant accumulation of anucleated cells (zoids). Therefore, the role of TbMCM-BP seems to be specific at least during cell cycle progression. It appears that TbMCM-BP is mostly chromatin-unbound because we were not able to immunoprecipitate much DNA with TbMCM-BP (data not shown) suggesting that TbMCM-BP plays its role in chromosome maintenance via transient interaction with chromatin or via indirect interaction with chromatin.

Topoisomerase 2α (Top2 in yeasts) is required for centromere maintenance and for replication termination [71]–[73]. Telomere entanglement in *taz1Δ* (a fission yeast homologue of TRF, a telomere-binding protein) can be relieved by a *top2* mutant [74]. In *Xenopus*, MCM-BP is required at the late S phase to remove MCM, probably to block re-replication [25]. Similarly, ICRF193, an inhibitor of Top2, inhibited MCM dissociation at the end of replication [25]. Interestingly, TbTOPO2α co-purified with TbMCM-BP (Table S2 and Table S3). As only four unique peptides of TbTOPO2α were identified by mass spectrometry, the interaction between TbTOPO2α and TbMCM-BP might occur transiently at a specific phase of the cell cycle. One possibility is that TbMCM-BP may be required for the coordination of replication termination and chromosome segregation by interacting with TOPO2α at the end of S-phase. Catenated sister chromosomes and/or entangled telomeres may induce modification of chromatin structure and influence transcriptional status of silent loci.

Derepression was much stronger at subtelomeric *VSG*s than at silent BES promoters. Therefore, TbMCM-BP may not have a direct effect on the activity of silent BES promoters. Transcription initiates at silent BESs but the transcripts are attenuated [43], thereby creating a gradient of levels of transcripts along silent BESs with less transcripts produced near telomeres. Disruption of telomere-binding protein TbRAP1 has a stronger effect on genes near telomeres in silent BESs [7]. RNAs from derepressed silent *VSG*s in TbMCM-BP-depleted cells seem to be polyadenylated, because they were readily identified in oligo-dT-reverse transcribed cDNA, and VSGs expressed from silent BESs were detected by immunoblot and IFs. We do not know whether the role of TbMCM-BP in *VSG* silencing is dependent on TbRAP1, but it is possible that TbMCM-BP functions with some chromatin binding proteins with greater affinities to telomeres or subtelomeric regions. Chromatin changes along silent BESs may facilitate transcription elongation, leading to a disproportional increase of transcripts from the most distally positioned *VSG* gene.

Depletion of TbMCM-BP also increased the levels of procyclin and *PAG* RNAs, which are transcribed from an RNA pol I promoter that is repressed in BF. This transcription unit is located between two convergent RNA Pol II polycistrons. Chromatin remodellers, TbISWI and TbNLP, are also required for repression of procyclin and *PAG*s [64], [65]. Therefore, TbMCM-BP seems to work in a separate pathway from TbISWI and/or TbNLP regarding the silencing of *VSG* loci, but might work together with these chromatin factors in silencing of a chromosome-internally located RNA Pol I-transcription unit. Depletion of TbCAF-1b led to derepression of silent *VSG*s specifically in late-S phase and G2 [13] and depletion of TbSPT16 caused G2/M-specific derepression of silent BES promoters [12]. However, derepression of silent *VSG*s was observed during all cell cycle in TbASF1A-depleted cells [13]. TbMCM-BP is required for S/G2 cell cycle progression, but silent *VSG* derepression does not seem to depend on the phase of the cell cycle, or on abnormalities in cell cycle. However, we were able to detect silent VSG expression only in a subset of TbMCM-BP-depleted cells by IF, and a more quantitative method would be necessary to firmly determine if there is a correlation between TbMCM-BP-mediated *VSG* silencing and cell cycle. Collectively, data from various genes suggest that networks of multiple complex pathways are involved in silencing *VSG* and procyclin transcription loci in BF trypanosomes. It has been difficult to perform complicated genetic analysis in *T. brucei*, partly because generation of mutations has relied solely on sequential transfections using limited number of selection markers, due to inability to reproduce meiotic stages in culture. With our loxP-Cre and conditional KO systems, we can now analyze, relatively easily, genetic interactions between multiple key factors by creating double or triple mutants and this will be helpful to get more detailed molecular understandings on complex mechanisms, such as antigenic variation.

This is the first time that a *VSG* silencing factor has been selected by a phenotype-based large-scale screening approach, and *T. brucei* is the first organism in which MCM-BP was identified in such way. TbMCM-BP is strongly associated with four *T. brucei* MCM-subunits, MCM4-MCM7, and MCM8, a subunit that is uniquely co-purified only with MCM-BP in *T. brucei*. Whether or not this TbMCM-BP's unique composition has special roles in antigenic variation in *T. brucei* remains to be determined.

## Materials and Methods

### *Trypanosome* strains

*Trypanosoma brucei* bloodstream forms (strain Lister 427 antigenic type MITat1.2 clone 221a) were cultured in HMI-9 at 37°C [75]. The cell lines constructed for this study are listed in Table S4, and bloodstream form (BF) trypanosomes were either wild type or of ‘single marker’ (SM) background expressing T7 RNA polymerase and Tet repressor (TetR) [45]. Procyclic trypanosomes (PF427) were from the same Lister 427 strain and cultured in SDM-79 at 27°C [76]. Stable clones of BF and PF trypanosomes were in HMI-9 and SDM-79 media, respectively. BF clones were maintained in HMI-9 media containing necessary antibiotics at the following concentrations, unless otherwise stated: 2.5 µg/ml G418 (Sigma); 5 µg/ml blasticidin (Invivogen); 5 µg/ml hygromycin (Sigma); 0.1 µg/ml puromycin (Sigma); 1 µg/ml phleomycin (Invivogen). Stable clones of procyclic trypanosomes were obtained using electroporation and maintained in SDM-79 media containing necessary antibiotics at the following concentrations: 15 µg/ml G418 (Sigma); 40 µg/ml hygromycin (Sigma); 1 µg/ml puromycin (Sigma).

Triple-reporter cell line (HSTB-10): PF427 was transfected with a linearized plasmid (pHJ1) containing a puromycin-resistance (*PUR*), luciferase (*LUC*), and emerald-GFP (em*GFP*) genes, and targeting sequences immediate downstream of a BES promoter. The targeted BES was identified by PCR analyses using a primer set specific to the insert and *ESAG*7 located downstream of a BES promoter and using a polymorphism of *ESAG*6 [77]. The clone that had the reporter insertion at the BES11 (HSTB-10) was obtained.

Reporter cell line expressing the *mariner* transposase (HSTB-39): The HSTB-10 was transfected with a pHJ2 to stably express *mariner* transposase.

*TbMCM-BP* knock out (KO) cell lines: one allele of *TbMCM-BP* was deleted (HSTB-317) using *PUR* conjugated with *Herpes simplex virus* thymidine kinase gene (*HSVTK* or *TK*) flanked by loxP sites so that the markers can be removed by expressing Cre-recombinase and be reused [78]. To delete the second allele, a fragment of pSY45 digested with Sal I and Not I containing 650 bp upstream homology sequences, *HYG-TK*, and 1042 bp downstream homology sequences were transfected either into wild type or HSTB-317.

*TbMCM-BP* RNAi cell lines (HSTB-464 and -465): the wild-type *MCM-BP* allele in the sKO (HSTB-317) was epitope-tagged *in situ* with 3xmyc using one-step PCR epitope tagging plasmid pMOTag53M [79] to make HSTB-459. The HSTB-459 was transfected with pHJ35 containing 437 bp RNAi sequences specific to *TbMCM-BP* to make HSTB-464 and -465.

*TbMCM-BP* conditional knock out (cKO) cell line (HSTB-660): HSTB-317 (*TbMCM-BP* sKO, *mcm-BPΔPUR-TK/MCM-BP-wt*) was transfected with pLEW100-Cre-EP1 [78] to make inducible Cre expression cell line (HSTB-645). The *PUR-TK* marker was removed by expressing Cre recombinase to obtain HSTB-647. The wild type allele of *TbMCM-BP* in the HSTB-647 was then replaced with a *TbMCM-BP* cKO cassette containing *MCM-BP-myc* followed by *HYG-TK* and flanked by loxP sites (pDS24), to make HSTB-660.

*TbMCM-BP* cKO cell line with a silent BES marked with a triple reporter (HSTB-683 and -684): HSTB-660 was transfected with pHJ1 to randomly target one of silent BESs with markers, *PUR*-*LUC*-em*GFP*. Targeting was confirmed by PCR analyses and puromycin sensitivity at 10 µg/ml.

To make a PF cell line expressing TbMCM-BP-PTP, HSTB-586 (*mcm-BPΔHYG-TK/MCM-BP*) was transfected with pDS15 (pMCM-BP-PTP-NEO). The tag sequence was fused to the C-terminus of an endogenous *TbMCM-BP* allele by targeted insertion of the plasmid pDS15. Cells expressing MCM-BP-3xmyc, MCM2-flag, and MCM5-flag were constructed using one-step PCR epitope tagging method [79]. The sequences of primers used here are available upon request.

### Construction of plasmids

Plasmids used for this study are listed in Table S5. Detailed construction information and maps are available upon request.

### Loss-of-silencing (LOS) screen

To make a loss-of-silencing reporter cell line (HSTB-10), PF trypanosomes were transfected with a pHJ1, a triple-reporter plasmid containing a puromycin-resistance (*PUR*), luciferase (*LUC*), and emerald-GFP (em*GFP*) genes, and targeting sequences immediate downstream of a BES promoter. The HSTB-10 was transfected with a pHJ2 to stably express *mariner* transposase to make HSTB-39. We titrated puromycin concentration for the screen and confirmed that the 100 µg/ml effectively kills the reporter cells with the rate of spontaneous mutation ∼3×10^−7^. Random insertional mutagenesis was induced by transfecting cells with the donor-plasmid pSGL35 [44] containing a hygromycin-resistance gene (*HYG*) flanked by *mariner* inverted repeats (IRs) and a neomycin-resistance gene (*NEO*) to select cells transformed by the plasmid. The plasmid-transfected cells were expanded under G418 selection until the population reached 5×10^8^ and were distributed in the 96-well plates containing 100 µg/ml puromycin and 40 µg/ml hygromycin. 19 *PUR^R^ HYG^R^* clones were isolated and examined for luciferase activity (Promega). The transposon target sites were mapped by inverse-PCR followed by sequencing [44].

### mRNA preparation and reverse-transcription-quantitative real-time PCR

Total mRNA was prepared using RNA STAT60 (TEL-Test) as described in manufacturer's protocol and cDNAs were generated using oligo dT_20_ and reverse-transcriptase (Stratagene). RNA was amplified using primers specific to individual genes by quantitative PCR using the LightCycler 480 (Roche). Amplified double-stranded DNA product during 40 cycles was detected by SYBR Green I. All measurements were in triplicate and compared with a 1000-fold range of serially diluted standard genomic DNA prepared from the wild-type strain. The sequences of primers are available upon request.

### Co-immunoprecipitation and immunoblot

About 10^8^ cells were lysed in lysis buffer (25 mM Tris–HCl (pH 8.0), 1 mM EDTA, 0.5% NP-40, 10% glycerol, 1 mM phenylmethylsulfonylfloride (PMSF), 1 mM dithiothreitol (DTT), protease inhibitor cocktails (Sigma)). Cell lysates were immunoprecipitated and analyzed by immunoblot using with anti-myc (Sigma and in-house monoclonal antibody core facility) or anti-flag antibodies (Sigma). TAP immunoblot monitoring of TbMCM-BP-PTP was carried out with the monoclonal anti-protein C antibody HPC4 (Roche).

### Tandem affinity purification of TbMCM-BP and identification of co-purified proteins by mass spectrometry

Tandem affinity purification of TbMCM-BP-PTP was performed exactly as described in the standard protocol [63]. Purified proteins were separated on an SDS-10 to 20% polyacrylamide gradient gel and first stained with Sypro Ruby (Invitrogen) and then with Coomassie blue (Gelcode Coomassie stain; Pierce) according to the manufacturers' protocols. For the identification of co-purified proteins, the gel lane of the final eluate was cut into 15 pieces, and peptides derived from trypsin-digested proteins were separated by liquid chromatography and analyzed by tandem mass spectrometry using an Ultimate 3000 HPLC system (Dionex) and a nanospray LTQ Orbitrap XL (Thermo Scientific) mass spectrometer. Proteins were identified using Mascot and NCBI non-redundant protein sequence database of eukaryotes, with carbamidomethyl (C) as static and oxidation (M) as variable modifications. Peptide Mass tolerance was set to ±25 ppm and fragment mass tolerance to ±0.8 Da. Peptides were considered as identified when their score was ≥22 and expectation values were ≤0.05.

### Immunofluorescence and DNA staining

Immunofluorescence was carried out as described previously [80] with minor modifications. Briefly, cells were fixed with 1% paraformaldehyde for 5 minutes and permeabilized with 0.2% NP40 in PBS. Cells were incubated with chicken anti-VSG2 and either rabbit anti-VSG3 or VSG13, and then incubated with secondary antibodies (Alexa 488 for chicken and Texas Red for rabbit antibodies). TbMCM-BP-PTP was detected with a polyclonal anti-protein A antibody (Sigma) followed by an Alexa 594-conjugated anti-rabbit secondary antibody (Invitrogen). DNA was stained with 0.5 µg/ml DAPI. Images were captured using a Zeiss Axioplan 2 or AxioVert 200 fluorescence microscope and edited with Adobe Photoshop.

### Flow cytometry

Cells were fixed with 70% ice-cold ethanol and stained with 0.5 µg/ml propidium iodide to examine cell cycle progression by flow cytometry.

## Supporting Information

Figure S1 **Depletion of TbMCM-BP by RNAi caused cell growth defect.** The *TbMCM-BP^−/MYC^* strain was transfected with a construct containing an RNAi cassette specific to *TbMCM-BP*, which can be induced by adding tetracycline. Depletion of TbMCM-BP by RNAi showed a growth defect, which correlated with TbMCM-BP-myc disappearance. Two independent cell lines were examined and tubulin was used as a loading control. * indicates breakdown product of tubulin.
(TIF)

Figure S2 **TbMCM-BP deficiency accumulated anucleated (zoid) cells and abnormalities in cell cycle and division.** (A) Profiling of abnormal cells by DAPI staining. Anucleated cells greatly accumulated upon *TbMCM-BP* removal. Other abnormalities observed less frequently are categorized. (B) Examples of DAPI stained cells with the DNA content of 1N1K, 1N2K, and 2N2K, and of abnormal NK contents.
(TIF)

Table S1 **Mapping of transposon-targeted regions in** ***LOS*** **clones.**
(DOC)

Table S2 **Mass spectrometric identification of non-MCM proteins that co-purified with TbMCM-BP.**
(DOC)

Table S3 **Unique peptide sequences identified in each protein.** Note: Some cases, same unique peptide sequence contained redundant peptide which show(s) different methionine oxidation site (underlined).
(DOC)

Table S4 ***Trypanosoma brucei*** **strains used in this study.**
(DOC)

Table S5 **Plasmids used in this study.**
(DOC)

## References

[1] Horn D, Barry JD (2005) The central roles of telomeres and subtelomeres in antigenic variation in African trypanosomes. Chrom Res 13: 525–533. 16132817 10.1007/s10577-005-0991-8

[2] Günzl A, Bruderer T, Laufer G, Schimanski B, Tu LC, et al (2003) RNA polymerase I transcribes procyclin genes and variant surface glycoprotein gene expression sites in *Trypanosoma brucei* . Eukaryot Cell 2: 542–551. 12796299 10.1128/EC.2.3.542-551.2003 PMC161450

[3] Hertz-Fowler C, Figueiredo LM, Quail MA, Becker M, Jackson A, et al (2008) Telomeric expression sites are highly conserved in *Trypanosoma brucei* . PLoS ONE 3: e3527. 18953401 10.1371/journal.pone.0003527 PMC2567434

[4] Alarcon CM, Son HJ, Hall T, Donelson JE (1994) A monocistronic transcript for a trypanosome variant surface glycoprotein. Mol Cell Biol 14: 5579–5591. 8035832 10.1128/mcb.14.8.5579-5591.1994 PMC359077

[5] Nagoshi YL, Alarcon CM, Donelson JE (1995) The putative promoter for a metacyclic VSG gene in African trypanosomes. Mol Biochem Parasitol 72: 33–45. 8538698 10.1016/0166-6851(95)00062-6

[6] Alsford S, Kawahara T, Isamah C, Horn D (2007) A sirtuin in the African trypanosome is involved in both DNA repair and telomeric gene silencing but is not required for antigenic variation. Mol Microbiol 63: 724–736. 17214740 10.1111/j.1365-2958.2006.05553.x

[7] Yang X, Figueiredo LM, Espinal A, Okubo E, Li B (2009) RAP1 is essential for silencing telomeric variant surface glycoprotein genes in *Trypanosoma brucei* . Cell 137: 99–109. 19345190 10.1016/j.cell.2009.01.037 PMC2673096

[8] Hughes K, Wand M, Foulston L, Young R, Harley K, et al (2007) A novel ISWI is involved in VSG expression site downregulation in African trypanosomes. EMBO J 26: 2400–2410. 17431399 10.1038/sj.emboj.7601678 PMC1864976

[9] Figueiredo LM, Janzen CJ, Cross GAM (2008) A histone methyltransferase modulates antigenic variation in African trypanosomes. PLoS Biol 6: e161. 18597556 10.1371/journal.pbio.0060161 PMC2443197

[10] Kawahara T, Siegel TN, Ingram AK, Alsford S, Cross GAM, et al (2008) Two essential MYST-family proteins display distinct roles in histone H4K10 acetylation and telomeric silencing in trypanosomes. Mol Microbiol 69: 1054–1068. 18631159 10.1111/j.1365-2958.2008.06346.x PMC2556858

[11] Wang QP, Kawahara T, Horn D (2010) Histone deacetylases play distinct roles in telomeric VSG expression site silencing in African trypanosomes. Mol Microbiol 77: 1237–1245. 20624217 10.1111/j.1365-2958.2010.07284.x PMC2941730

[12] Denninger V, Fullbrook A, Bessat M, Ersfeld K, Rudenko G (2010) The FACT subunit TbSpt16 is involved in cell cycle specific control of VSG expression sites in *Trypanosoma brucei* . Mol Microbiol 78: 459–474. 20879999 10.1111/j.1365-2958.2010.07350.x PMC3034197

[13] Alsford S, Horn D (2012) Cell-cycle-regulated control of VSG expression site silencing by histones and histone chaperones ASF1A and CAF-1b in *Trypanosoma brucei* . Nucl Acids Res 40: 10150–10160. 22941664 10.1093/nar/gks813 PMC3488249

[14] Povelones ML, Gluenz E, Dembek M, Gull K, Rudenko G (2012) Histone H1 Plays a Role in Heterochromatin Formation and VSG Expression Site Silencing in *Trypanosoma brucei* . PLoS Pathog 8: e1003010. 23133390 10.1371/journal.ppat.1003010 PMC3486875

[15] Sakwe AM, Nguyen T, Athanasopoulos V, Shire K, Frappier L (2007) Identification and characterization of a novel component of the human minichromosome maintenance complex. Mol Cell Biol 27: 3044–3055. 17296731 10.1128/MCB.02384-06 PMC1899943

[16] Ding L, Forsburg SL (2011) *Schizosaccharomyces pombe* Minichromosome Maintenance-binding Protein (MCM-BP) Antagonizes MCM Helicase. J Biol Chem 286: 32918–32930. 21813639 10.1074/jbc.M111.282541 PMC3190919

[17] Takahashi N, Quimbaya M, Schubert V, Lammens T, Vandepoele K, et al (2010) The MCM-binding protein ETG1 aids sister chromatid cohesion required for postreplicative homologous recombination repair. PLoS Genet 6: e1000817. 20090939 10.1371/journal.pgen.1000817 PMC2806904

[18] Pospiech H, Grosse F, Pisani FM (2010) The initiation step of eukaryotic DNA replication. Subcell Biochem 50: 79–104. 20012578 10.1007/978-90-481-3471-7_5

[19] Parrilla-Castellar ER, Arlander SJ, Karnitz L (2004) Dial 9-1-1 for DNA damage: the Rad9-Hus1-Rad1 (9-1-1) clamp complex. DNA repair 3: 1009–1014. 15279787 10.1016/j.dnarep.2004.03.032

[20] Johnson A, O'Donnell M (2005) Cellular DNA replicases: components and dynamics at the replication fork. Annu Rev Biochem 74: 283–315. 15952889 10.1146/annurev.biochem.73.011303.073859

[21] Majka J, Burgers PM (2004) The PCNA-RFC families of DNA clamps and clamp loaders. Prog Nucl Acid Res Mol Biol 78: 227–260. 10.1016/S0079-6603(04)78006-X 15210332

[22] Mayer ML, Gygi SP, Aebersold R, Hieter P (2001) Identification of RFC(Ctf18p, Ctf8p, Dcc1p): an alternative RFC complex required for sister chromatid cohesion in *S. cerevisiae* . Mol Cell 7: 959–970. 11389843 10.1016/s1097-2765(01)00254-4

[23] Forsburg SL (2004) Eukaryotic MCM proteins: beyond replication initiation. Microbiol Mol Biol Rev 68: 109–131. 15007098 10.1128/MMBR.68.1.109-131.2004 PMC362110

[24] Takahashi N, Lammens T, Boudolf V, Maes S, Yoshizumi T, et al (2008) The DNA replication checkpoint aids survival of plants deficient in the novel replisome factor ETG1. EMBO J 27: 1840–1851. 18528439 10.1038/emboj.2008.107 PMC2486427

[25] Nishiyama A, Frappier L, Mechali M (2011) MCM-BP regulates unloading of the MCM2-7 helicase in late S phase. Genes Dev 25: 165–175. 21196493 10.1101/gad.614411 PMC3022262

[26] Shareef MM, King C, Damaj M, Badagu R, Huang DW, et al (2001) Drosophila heterochromatin protein 1 (HP1)/origin recognition complex (ORC) protein is associated with HP1 and ORC and functions in heterochromatin-induced silencing. Mol Biol Cell 12: 1671–1685. 11408576 10.1091/mbc.12.6.1671 PMC37332

[27] Prasanth SG, Shen Z, Prasanth KV, Stillman B (2010) Human origin recognition complex is essential for HP1 binding to chromatin and heterochromatin organization. Proc Natl Acad Sci U S A 107: 15093–15098. 20689044 10.1073/pnas.1009945107 PMC2930523

[28] Mancio-Silva L, Rojas-Meza AP, Vargas M, Scherf A, Hernandez-Rivas R (2008) Differential association of Orc1 and Sir2 proteins to telomeric domains in *Plasmodium falciparum* . J Cell Sci 121: 2046–2053. 18525026 10.1242/jcs.026427

[29] Fox CA, Ehrenhofermurray AE, Loo S, Rine J (1997) The origin recognition complex, SIR1, and the S phase requirement for silencing. Science 276: 1547–1551. 9171055 10.1126/science.276.5318.1547

[30] Ishimi Y, Ichinose S, Omori A, Sato K, Kimura H (1996) Binding of human minichromosome maintenance proteins with histone H3. J Biol Chem 271: 24115–24122. 8798650 10.1074/jbc.271.39.24115

[31] Snyder M, He W, Zhang JJ (2005) The DNA replication factor MCM5 is essential for Stat1-mediated transcriptional activation. Proc Natl Acad Sci U S A 102: 14539–14544. 16199513 10.1073/pnas.0507479102 PMC1253610

[32] DaFonseca CJ, Shu F, Zhang JJ (2001) Identification of two residues in MCM5 critical for the assembly of MCM complexes and Stat1-mediated transcription activation in response to IFN-gamma. Proc Natl Acad Sci U S A 98: 3034–3039. 11248027 10.1073/pnas.061487598 PMC30602

[33] Holland L, Gauthier L, Bell-Rogers P, Yankulov K (2002) Distinct parts of minichromosome maintenance protein 2 associate with histone H3/H4 and RNA polymerase II holoenzyme. Eur J Biochem 269: 5192–5202. 12392551 10.1046/j.1432-1033.2002.03224.x

[34] Dziak R, Leishman D, Radovic M, Tye BK, Yankulov K (2003) Evidence for a role of MCM (mini-chromosome maintenance)5 in transcriptional repression of sub-telomeric and Ty-proximal genes in *Saccharomyces cerevisiae* . J Biol Chem 278: 27372–27381. 12750362 10.1074/jbc.M301110200

[35] Godoy PD, Nogueira-Junior LA, Paes LS, Cornejo A, Martins RM, et al (2009) Trypanosome prereplication machinery contains a single functional orc1/cdc6 protein, which is typical of archaea. Eukaryot Cell 8: 1592–1603. 19717742 10.1128/EC.00161-09 PMC2756867

[36] Tiengwe C, Marcello L, Farr H, Gadelha C, Burchmore R, et al (2012) Identification of ORC1/CDC6-Interacting Factors in *Trypanosoma brucei* Reveals Critical Features of Origin Recognition Complex Architecture. PLoS ONE 7: e32674. 22412905 10.1371/journal.pone.0032674 PMC3297607

[37] Dang HQ, Li Z (2011) The Cdc45.Mcm2-7.GINS protein complex in trypanosomes regulates DNA replication and interacts with two Orc1-like proteins in the origin recognition complex. J Biol Chem 286: 32424–32435. 21799014 10.1074/jbc.M111.240143 PMC3173170

[38] Tiengwe C, Marcello L, Farr H, Dickens N, Kelly S, et al (2012) Genome-wide analysis reveals extensive functional interaction between DNA replication initiation and transcription in the genome of *Trypanosoma brucei* . Cell reports 2: 185–197. 22840408 10.1016/j.celrep.2012.06.007 PMC3607257

[39] Benmerzouga I, Concepcion-Acevedo J, Kim HS, Vandoros AV, Cross GAM, et al (2013) *Trypanosoma brucei Orc1* is essential for nuclear DNA replication and affects both VSG silencing and VSG switching. Mol Microbiol 87: 196–210. 23216794 10.1111/mmi.12093 PMC3535549

[40] Kim HS, Cross GAM (2010) TOPO3alpha influences antigenic variation by monitoring expression-site-associated VSG switching in *Trypanosoma brucei* . PLoS Pathog 6: e1000992. 20628569 10.1371/journal.ppat.1000992 PMC2900300

[41] Kim HS, Cross GAM (2011) Identification of *Trypanosoma brucei* RMI1/BLAP75 homologue and its roles in antigenic variation. PLoS ONE 6: e25313. 21980422 10.1371/journal.pone.0025313 PMC3182221

[42] Peacock L, Ferris V, Sharma R, Sunter J, Bailey M, et al (2011) Identification of the meiotic life cycle stage of *Trypanosoma brucei* in the tsetse fly. Proc Natl Acad Sci U S A 108: 3671–3676. 21321215 10.1073/pnas.1019423108 PMC3048101

[43] Vanhamme L, Poelvoorde P, Pays A, Tebabi P, Xong HV, et al (2000) Differential RNA elongation controls the variant surface glycoprotein gene expression sites of *Trypanosoma brucei* . Mol Microbiol 36: 328–340. 10792720 10.1046/j.1365-2958.2000.01844.x

[44] Leal S, Acosta-Serrano A, Morris JC, Cross GAM (2004) Transposon mutagenesis of *Trypanosoma brucei* identifies glycosylation mutants resistant to Concanavalin A. J Biol Chem 279: 28979–28988. 15123607 10.1074/jbc.M403479200

[45] Wirtz E, Leal S, Ochatt C, Cross GAM (1999) A tightly regulated inducible expression system for dominant negative approaches in *Trypanosoma brucei* . Mol Biochem Parasitol 99: 89–101. 10215027 10.1016/s0166-6851(99)00002-x

[46] Boothroyd CE, Dreesen O, Leonova T, Ly KI, Figueiredo LM, et al (2009) A yeast-endonuclease-generated DNA break induces antigenic switching in *Trypanosoma brucei* . Nature 459: 278–281. 19369939 10.1038/nature07982 PMC2688456

[47] Wickstead B, Ersfeld K, Gull K (2004) The small chromosomes of *Trypanosoma brucei* involved in antigenic variation are constructed around repetitive palindromes. Genome Res 14: 1014–1024. 15173109 10.1101/gr.2227704 PMC419779

[48] Horn D, McCulloch R (2010) Molecular mechanisms underlying the control of antigenic variation in African trypanosomes. Curr Opin Microbiol 13: 700–705. 20884281 10.1016/j.mib.2010.08.009 PMC3117991

[49] Horn D, Cross GAM (1997) Position-dependent and promoter-specific regulation of gene expression in *Trypanosoma brucei* . EMBO J 16: 7422–7431. 9405371 10.1093/emboj/16.24.7422 PMC1170342

[50] Horn D, Cross GAM (1995) A developmentally regulated position effect at a telomeric locus in *Trypanosoma brucei* . Cell 83: 555–561. 7585958 10.1016/0092-8674(95)90095-0

[51] Biebinger S, Rettenmaier S, Flaspohler J, Hartmann C, Penadiaz J, et al (1996) The PARP promoter of *Trypanosoma brucei* is developmentally regulated in a chromosomal context. Nucl Acids Res 24: 1202–1211. 8614620 10.1093/nar/24.7.1202 PMC145797

[52] Jagannathan M, Sakwe AM, Nguyen T, Frappier L (2012) The MCM-associated protein MCM-BP is important for human nuclear morphology. J Cell Sci 125: 133–143. 22250201 10.1242/jcs.089938

[53] Ploubidou A, Robinson DR, Docherty RC, Ogbadoyi EO, Gull K (1999) Evidence for novel cell cycle checkpoints in trypanosomes: kinetoplast segregation and cytokinesis in the absence of mitosis. Journal of Cell Science 112: 4641–4650. 10574712 10.1242/jcs.112.24.4641

[54] Cross GAM, Manning JC (1973) Cultivation of *Trypanosoma brucei* sspp. in semi-defined and defined media. Parasitology 67: 315–331. 4761771 10.1017/s0031182000046540

[55] Robinson DR, Sherwin T, Ploubidou A, Byard EH, Gull K (1995) Microtubule polarity and dynamics in the control of organelle positioning, segregation, and cytokinesis in the trypanosome cell cycle. J Cell Biol 128: 1163–1172. 7896879 10.1083/jcb.128.6.1163 PMC2120423

[56] Hammarton TC, Clark J, Douglas F, Boshart M, Mottram JC (2003) Stage-specific differences in cell cycle control in *Trypanosoma brucei* revealed by RNA interference of a mitotic cyclin. J Biol Chem 278: 22877–22886. 12682070 10.1074/jbc.M300813200

[57] Li Z, Wang C (2006) Changing roles of aurora-B kinase in two life cycle stages of *Trypanosoma brucei* . Eukaryot Cell 5: 1026–1035. 16835447 10.1128/EC.00129-06 PMC1489291

[58] Tu X, Wang C (2005) Pairwise knockdowns of cdc2-related kinases (CRKs) in *Trypanosoma brucei* identified the CRKs for G1/S and G2/M transitions and demonstrated distinctive cytokinetic regulations between two developmental stages of the organism. Eukaryot Cell 4: 755–764. 15821135 10.1128/EC.4.4.755-764.2005 PMC1087811

[59] Umeyama T, Wang CC (2008) Polo-like kinase is expressed in S/G2/M phase and associated with the flagellum attachment zone in both procyclic and bloodstream forms of *Trypanosoma brucei* . Eukaryot Cell 7: 1582–1590. 18621923 10.1128/EC.00150-08 PMC2547065

[60] Hammarton TC, Kramer S, Tetley L, Boshart M, Mottram JC (2007) *Trypanosoma brucei* Polo-like kinase is essential for basal body duplication, kDNA segregation and cytokinesis. Mol Microbiol 65: 1229–1248. 17662039 10.1111/j.1365-2958.2007.05866.x PMC2169650

[61] Li B, Espinal A, Cross GAM (2005) Trypanosome telomeres are protected by a homologue of mammalian TRF2. Mol Cell Biol 25: 5011–5021. 15923618 10.1128/MCB.25.12.5011-5021.2005 PMC1140576

[62] Li JJ, Schnick J, Hayles J, MacNeill SA (2011) Purification and functional inactivation of the fission yeast MCM(MCM-BP) complex. FEBS Lett 585: 3850–3855. 22036784 10.1016/j.febslet.2011.10.033

[63] Schimanski B, Nguyen TN, Günzl A (2005) Highly efficient tandem affinity purification of trypanosome protein complexes based on a novel epitope combination. Eukaryot Cell 4: 1942–1950. 16278461 10.1128/EC.4.11.1942-1950.2005 PMC1287860

[64] Stanne TM, Kushwaha M, Wand M, Taylor JE, Rudenko G (2011) TbISWI regulates multiple polymerase I (Pol I)-transcribed loci and is present at Pol II transcription boundaries in *Trypanosoma brucei* . Eukaryot Cell 10: 964–976. 21571922 10.1128/EC.05048-11 PMC3147422

[65] Narayanan MS, Kushwaha M, Ersfeld K, Fullbrook A, Stanne TM, et al (2011) NLP is a novel transcription regulator involved in VSG expression site control in *Trypanosoma brucei* . Nucl Acids Res 39: 2018–2031. 21076155 10.1093/nar/gkq950 PMC3064810

[66] Gueiros-Filho FJ, Beverley SM (1996) Selection against the dihydrofolate reductase-thymidylate synthase (DHFR-TS) locus as a probe of genetic alterations in *Leishmania major* . Mol Cell Biol 16: 5655–5663. 8816478 10.1128/mcb.16.10.5655 PMC231565

[67] Landeira D, Bart JM, Van Tyne D, Navarro M (2009) Cohesin regulates VSG monoallelic expression in trypanosomes. J Cell Biol 186: 243–254. 19635842 10.1083/jcb.200902119 PMC2717648

[68] Sheader K, Vruchte DT, Rudenko G (2004) Bloodstream form-specific up-regulation of silent VSG expression sites and procyclin in *Trypanosoma brucei* after inhibition of DNA synthesis or DNA damage. J Biol Chem 279: 13363–13374. 14726511 10.1074/jbc.M312307200

[69] Glover TW, Berger C, Coyle J, Echo B (1984) DNA polymerase alpha inhibition by aphidicolin induces gaps and breaks at common fragile sites in human chromosomes. Hum Genet 67: 136–142. 6430783 10.1007/BF00272988

[70] Nguyen T, Jagannathan M, Shire K, Frappier L (2012) Interactions of the human MCM-BP protein with MCM complex components and Dbf4. PLoS ONE 7: e35931. 22540012 10.1371/journal.pone.0035931 PMC3335088

[71] Fachinetti D, Bermejo R, Cocito A, Minardi S, Katou Y, et al (2010) Replication termination at eukaryotic chromosomes is mediated by Top2 and occurs at genomic loci containing pausing elements. Mol Cell 39: 595–605. 20797631 10.1016/j.molcel.2010.07.024 PMC3041477

[72] Luo K, Yuan J, Chen J, Lou Z (2009) Topoisomerase IIalpha controls the decatenation checkpoint. Nat Cell Biol 11: 204–210. 19098900 10.1038/ncb1828 PMC2712943

[73] Nitiss JL (2009) DNA topoisomerase II and its growing repertoire of biological functions. Nature reviews 9: 327–337. 10.1038/nrc2608 PMC2730144 19377505

[74] Germe T, Miller K, Cooper JP (2009) A non-canonical function of topoisomerase II in disentangling dysfunctional telomeres. EMBO J 28: 2803–2811. 19680223 10.1038/emboj.2009.223 PMC2750024

[75] Hirumi H, Hirumi K (1989) Continuous cultivation of *Trypanosoma brucei* bloodstream forms in a medium containing a low concentration of serum protein without feeder cell layers. J Parasitol 75: 985–989. 2614608

[76] Brun R, Schonenberger M (1979) Cultivation and in vitro cloning of procyclic culture forms of *Trypanosoma brucei* in a semi-defined medium. Acta Tropica 36: 289–292. 43092

[77] Becker M, Aitcheson N, Byles E, Wickstead B, Louis E, et al (2004) Isolation of the repertoire of VSG expression site containing telomeres of *Trypanosoma brucei* 427 using transformation-associated recombination in yeast. Genome Res 14: 2319–2329. 15520294 10.1101/gr.2955304 PMC525691

[78] Scahill MD, Pastar I, Cross GAM (2008) CRE recombinase-based positive-negative selection systems for genetic manipulation in *Trypanosoma brucei* . Mol Biochem Parasitol 157: 73–82. 18006158 10.1016/j.molbiopara.2007.10.003 PMC2211722

[79] Oberholzer M, Morand S, Kunz S, Seebeck T (2006) A vector series for rapid PCR-mediated C-terminal in situ tagging of *Trypanosoma brucei* genes. Mol Biochem Parasitol 145: 117–120. 16269191 10.1016/j.molbiopara.2005.09.002

[80] Lowell JE, Cross GAM (2004) A variant histone H3 is enriched at telomeres in *Trypanosoma brucei* . J Cell Sci 117: 5937–5947. 15522895 10.1242/jcs.01515

