# Supplementary figures and images for: MCM-BP Is Required for Repression of Life-Cycle Specific Genes Transcribed by RNA Polymerase I in the Mammalian Infectious Form of Trypanosoma brucei
Source: PLoS One. 2013 Feb 25;8(2):e57001. doi: 10.1371/journal.pone.0057001 (PMC3581582; doi:10.1371/journal.pone.0057001)

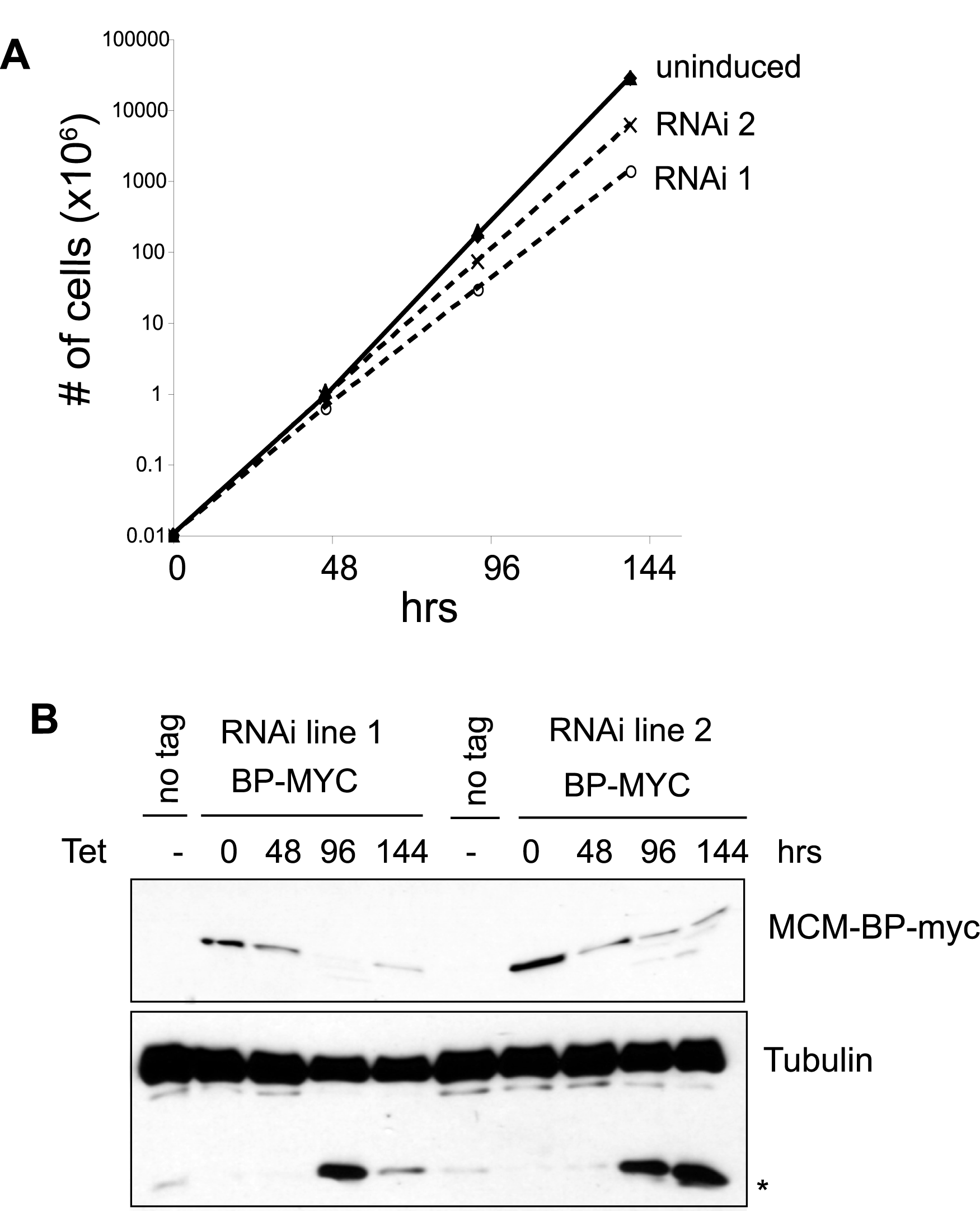

Supplement: Figure S1 — Depletion of TbMCM-BP by RNAi caused cell growth defect. The TbMCM-BP−/MYC strain was transfected with a construct containing an RNAi cassette specific to TbMCM-BP, which can be induced by adding tetracycline. Depletion of TbMCM-BP by RNAi showed a growth defect, which correlated with TbMCM-BP-myc disappearance. Two independent cell lines were examined and tubulin was used as a loading control. * indicates breakdown product of tubulin. (TIF) [file pone.0057001.s001.tif]

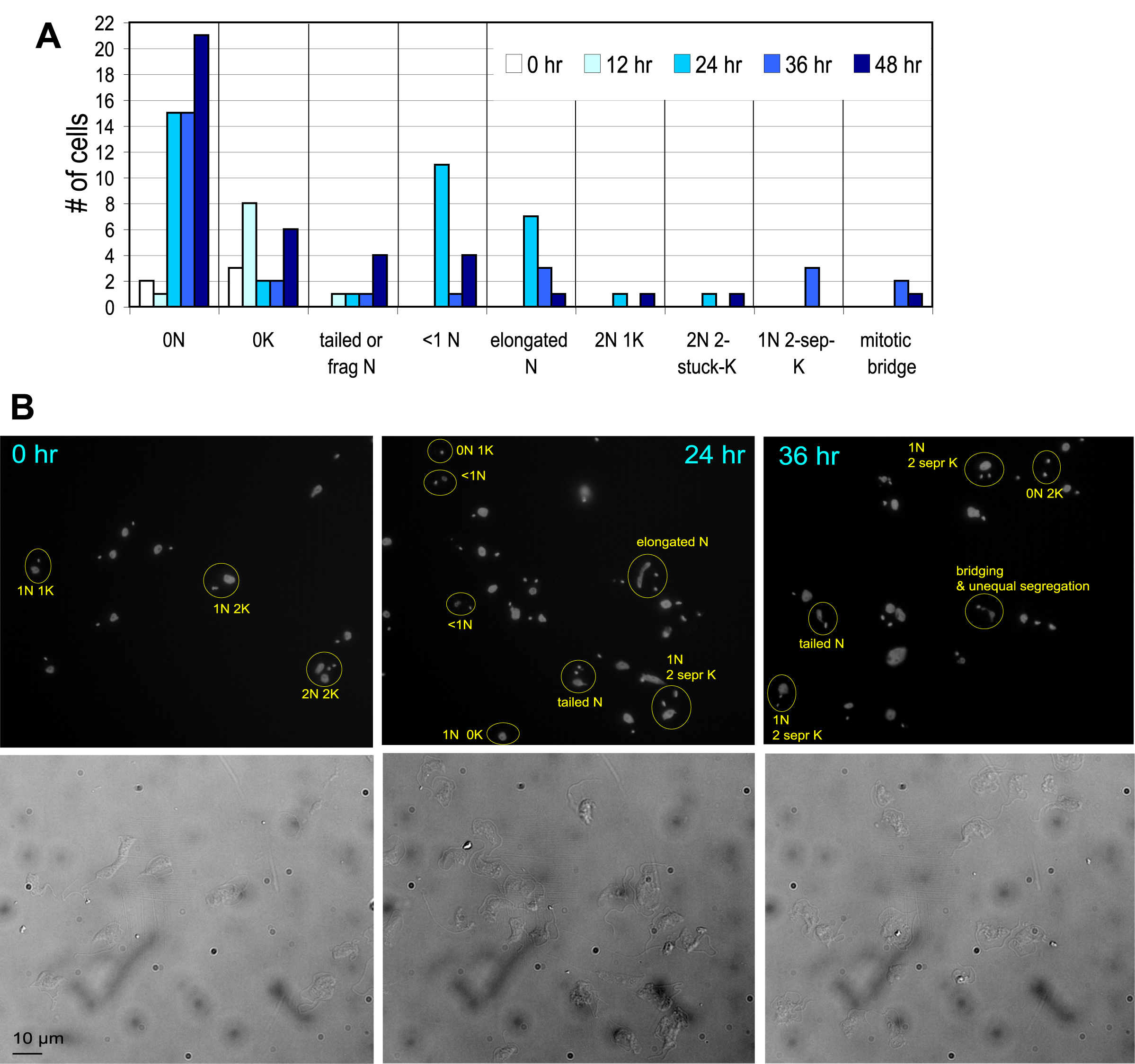

Supplement: Figure S2 — TbMCM-BP deficiency accumulated anucleated (zoid) cells and abnormalities in cell cycle and division. (A) Profiling of abnormal cells by DAPI staining. Anucleated cells greatly accumulated upon TbMCM-BP removal. Other abnormalities observed less frequently are categorized. (B) Examples of DAPI stained cells with the DNA content of 1N1K, 1N2K, and 2N2K, and of abnormal NK contents. (TIF) [file pone.0057001.s002.tif]
