# Supplementary material for: MCM-BP Is Required for Repression of Life-Cycle Specific Genes Transcribed by RNA Polymerase I in the Mammalian Infectious Form of Trypanosoma brucei
Source: PLoS One. 2013 Feb 25;8(2):e57001. doi: 10.1371/journal.pone.0057001 (PMC3581582; doi:10.1371/journal.pone.0057001)
Supplement: Table S1 — Mapping of transposon-targeted regions in LOS clones. (DOC) [file pone.0057001.s003.doc]

**Supporting Table S1.** Mapping of transposon-targeted regions in *LOS* clones

| # | Luciferase activity (Fold increase) | (Potential) target gene(s) | Targeted region |
| --- | --- | --- | --- |
| 1 | 3.9 | *Tb927.7.1770* | ORF |
| 2 | 2.4 | *Tb927.11.13130* (*TbMBAP1*, membrane-bound acid phosphatase 1 precursor) | ORF |
|  |  | *Tb927.4.5150* and *Tb927.4.5140* | Intergenic |
| 3 | 2.9 | *Tb927.7.1770* | ORF |
| 4 | 5.6 | *Tb927.7.1770* | ORF |
| 5 | 2.9 | *Tb927.7.320* (*TbRBP8*) | ORF |
|  |  | *Tb927.7.7180* | ORF |
| 6 | 2.9 | *Tb927.7.1770* | ORF |
| 7 | 4.8 | ? |  |
| 8 | 4.3 | *Tb927.7.1770* | ORF |
| 9 | 2.3 | *GRESAG4* array | Intergenic |
| 10 | 2.0 | *Tb927.4.4520* | Intergenic |
| 11 | 1.9 | *Tb927.11.15510* and *Tb927.11.15500* | Intergenic |
| 12 | 1.8 | *Tb927.7.920* (Putative dynein heavy chain) | ORF |
|  |  | Retrotransposon hotspot array | Intergenic |
| 13 | 2.7 | *Tb927.7.7180* | ORF |
| 14 | 1.6 | ? |  |
| 15 | 4.6 | Retrotransposon pseudogene array |  |
| 16 | 2.5 | *Tb927.9.15660* (*ESAG4*) | Intergenic |
| 17 | 1.6 | No ORF found within 10kb region |  |
| 18 | 2.0 | ? |  |
| 19 | 2.1 | *Tb927.7.7180* | ORF |
