# Supplementary material for: MCM-BP Is Required for Repression of Life-Cycle Specific Genes Transcribed by RNA Polymerase I in the Mammalian Infectious Form of Trypanosoma brucei
Source: PLoS One. 2013 Feb 25;8(2):e57001. doi: 10.1371/journal.pone.0057001 (PMC3581582; doi:10.1371/journal.pone.0057001)
Supplement: Table S2 — Mass spectrometric identification of non-MCM proteins that co-purified with TbMCM-BP. (DOC) [file pone.0057001.s004.doc]

**Supporting Table S2**. Mass spectrometric identification of non-MCM proteins that co-purified with TbMCM-BP

| Protein | Annotation^1^ | Mol. mass^2^ | # unique peptides^3^ | Sequence coverage^4^ | Peptide count^5^ | Best  ion score^6^ | Protein score^7^ |
| --- | --- | --- | --- | --- | --- | --- | --- |
| Putative DNA topoisomerase II | Tb927.11.11540 | 165.5 | 4 | 3% | 4 | 33 | 33 |
| ALBA3 | Tb927.4.2040 | 20.8 | 5 | 21% | 6 | 51 | 51 |
| Putative 40S ribosomal protein S4 | Tb927.11.3590 | 30.7 | 4 | 14% | 7 | 57 | 65 |
| Putative 40S ribosomal protein S3A | Tb927.10.3930 | 29.6 | 4 | 15% | 4 | 46 | 46 |
| Putative 40S ribosomal protein SA | Tb927.11.10790 | 27.8 | 3 | 15% | 3 | 44 | 44 |
| 60S ribosomal protein L2 | Tb927.5.1110 | 28.7 | 2 | 7% | 2 | 44 | 44 |
| Putative 60S ribosomal protein L5 | Tb927.9.15110 | 34.7 | 2 | 3% | 2 | 30 | 30 |
| Putative 40S ribosomal protein S18 | Tb927.10.5330 Tb927.10.5340 | 17.6 | 3 | 16% | 4 | 48 | 48 |
| Putative 60S acidic ribosomal subunit protein | Tb927.11.2050 | 34.9 | 5 | 17% | 7 | 38 | 42 |
| Putative chaperone protein DNAj | Tb927.2.5160 | 45.3 | 5 | 12% | 5 | 40 | 40 |
| Hypothetical conserved protein | Tb927.4.2640 | 146.5 | 2 | 1% | 5 | 36 | 39 |
| Putative RNA-binding protein | Tb927.6.3480 | 38.4 | 2 | 7% | 2 | 32 | 32 |
| Putative myosin heavy chain | Tb927.11.16310 | 121.5 | 3 | 3% | 3 | 29 | 29 |
| Putative 60S ribosomal protein L12 | Tb927.9.14000 | 24.3 | 1 | 5% | 1 | 39 | 39 |
| Hypothetical conserved protein | Tb927.4.3340 | 50.6 | 1 | 3% | 1 | 32 | 32 |
| Putative leucine-rich repeat protein | Tb927.6.1160 | 81.9 | 1 | 2% | 1 | 37 | 37 |
| Glyceraldehyde  3-phosphate dehydrogenase  glycosomal * | Tb927.6.4280 | 44.2 | 11 | 27% | 17 | 123 | 188 |
| Hypothetical  conserved protein * | Tb927.11.3510 | 92.3 | 3 | 9% | 3 | 18 | 33 |

^1^ Accession number of the GeneDB/TriTrypDB databases

^2^ Molecular mass in kDa.

^3^ Number of distinct peptide sequences identified for each protein

^4^ Maximal sequence coverage of unique peptides identified from a single gel slice

^5^ Total number of peptides identified for each co-purified protein

^6^ Highest ion score among identified peptides for each protein (ion scores were considered significant at ≥ 22 with expectation values ≤ 0.05)

^7^ Protein score is the sum of the highest ions score for each distinct sequence, corrected by Mascot to reduce the contribution of low-scoring potentially random matches.

* These are likely contaminants because these proteins co-purified with several other nuclear trypanosome complexes (data not shown).
