# Supplementary material for: MCM-BP Is Required for Repression of Life-Cycle Specific Genes Transcribed by RNA Polymerase I in the Mammalian Infectious Form of Trypanosoma brucei
Source: PLoS One. 2013 Feb 25;8(2):e57001. doi: 10.1371/journal.pone.0057001 (PMC3581582; doi:10.1371/journal.pone.0057001)
Supplement: Table S3 — Unique peptide sequences identified in each protein. Note: Some cases, same unique peptide sequence contained redundant peptide which show(s) different methionine oxidation site (underlined). (DOC) [file pone.0057001.s005.doc]

Supporting Table S3. Unique peptide sequences identified in each protein. Note: Some cases, same unique peptide sequence contained redundant peptide which show(s) different methionine oxidation site (underlined).

| **Protein** | **# Gel slice** |
| --- | --- |
| **MCM-BP (Tb927.7.1770)** | **5 and 6** |
| K.AAELGSAAAALSYVHR.H |  |
| K.GDSLLAEYILLHLCAR.V |  |
| K.LELPIDLSFLVLSTTK.L |  |
| K.LLAFSQSEPR.W |  |
| K.LTDEIGFLQLAVSVR.W |  |
| K.LTSSSLR.V |  |
| K.NTVCKGDSLLAEYILLHLCAR.V |  |
| K.QVLQLEYPYQK.L |  |
| K.RPLVLQHLK.N |  |
| R.AVAIASSGVQDAIFSAVHK.Q |  |
| R.CRGMIQEVDPSVALYR.A |  |
| R.DYFAQVR.R |  |
| R.EDDISTTQLSDK.L |  |
| R.EKAAELGSAAAALSYVHR.H |  |
| R.EVAPVGEVLLDLSK.L |  |
| R.FEREDDISTTQLSDK.L |  |
| R.GMIQEVDPSVALYR.A |  |
| R.GMIQEVDPSVALYR.A |  |
| R.IYSQPGINAIIR.E |  |
| [R.MTCLSWQR.V](http://ms-searching2.rockefeller.edu/mascot/cgi/peptide_view.pl?file=../data/20110304/F015199.dat&query=1254&hit=7&index=gi|72391014&px=1&section=5) |  |
| R.MTCLSWQR.V |  |
| R.TPMGDQTHQGEWNSEDPDVER.D |  |
| R.TPMGDQTHQGEWNSEDPDVERDPK.Q |  |
| R.VEGDIVNLDMWSAYMR.E |  |
| R.VEGDIVNLDMWSAYMR.E |  |
| R.VEGDIVNLDMWSAYMR.E |  |
| R.VEGDIVNLDMWSAYMR.E |  |
| [R.VITQEGGMPVGDLPLR.V](http://ms-searching2.rockefeller.edu/mascot/cgi/peptide_view.pl?file=../data/20110304/F015199.dat&query=4014&hit=1&index=gi|72391014&px=1&section=5) |  |
| R.VITQEGGMPVGDLPLR.V |  |
| R.VITQEGGMPVGDLPLRVEGDIVNLDMWSAYMR.E |  |
| R.VTSSLDEK.S |  |
| R.VYCQPDRPLCHFYFESK.R |  |
| R.WLPELTTEAAISNDISVDEVR.D |  |
|  |  |
| **MCM4 (Tb927.11.12250)** | **2 and 4** |
| K.ESQWNVNLNVVENLQIEPTLLSR.F |  |
| [K.SQLLTQVHEIAPR.G](http://ms-searching2.rockefeller.edu/mascot/cgi/peptide_view.pl?file=../data/20110304/F015212.dat&query=3775&hit=3&index=gi|74025442&px=1&section=5) |  |
| [R.APILLQHDGEVYLEGTEEKPYMPAR.V](http://ms-searching2.rockefeller.edu/mascot/cgi/peptide_view.pl?file=../data/20110304/F015212.dat&query=8918&hit=1&index=gi|74025442&px=1&section=5) |  |
| R.FLETFEMPQDVGAAPGADAGAHR.G |  |
| R.GHLATSR.A |  |
| R.GILLQLFGGTR.K |  |
| R.GLCCIDEFDK.M |  |
| R.GLCCIDEFDKMNEATR.S |  |
| R.GSQPAAVGQPQFFSR.R |  |
| R.HAFVEMTPLRPEPTDGSPTR.G |  |
| R.IFEPTR.C |  |
| R.INHNLSVFEDK.Q |  |
| R.INLDVFNAPDPTR.Q |  |
| R.LGDTVSVEDVR.E |  |
| R.LQEAPEHLADGDTPVTMSVVVYGDFVDSIVPGDR.V |  |
| R.LVAAAGTHPSMPGEDEFILR.I |  |
| [R.NAWADQQR.Q](http://ms-searching2.rockefeller.edu/mascot/cgi/peptide_view.pl?file=../data/20110304/F015212.dat&query=1423&hit=1&index=gi|74025442&px=1&section=5) |  |
| R.NFFLQELLR.L |  |
| R.NHPEITTLR.G |  |
| R.QLEQLVSLQGMVVR.V |  |
| [R.QLESMIR.L](http://ms-searching2.rockefeller.edu/mascot/cgi/peptide_view.pl?file=../data/20110304/F015212.dat&query=888&hit=2&index=gi|74025442&px=1&section=5) |  |
| R.QSAGEDEGLPEDPAVVAR.H |  |
| [R.SEINIILCGDPGVAK.S](http://ms-searching2.rockefeller.edu/mascot/cgi/peptide_view.pl?file=../data/20110304/F015212.dat&query=4217&hit=1&index=gi|74025442&px=1&section=5) |  |
| [R.SIFSTHVDAVHIEHR.R](http://ms-searching2.rockefeller.edu/mascot/cgi/peptide_view.pl?file=../data/20110304/F015212.dat&query=4910&hit=1&index=gi|74025442&px=1&section=5) |  |
| [R.SLFEMDLQLFCR.A](http://ms-searching2.rockefeller.edu/mascot/cgi/peptide_view.pl?file=../data/20110304/F015212.dat&query=4155&hit=1&index=gi|74025442&px=1&section=5) |  |
| R.SVLHEVMEQQTLSIAK.A |  |
| R.TIWGNEDVK.R |  |
| R.TIWGNEDVKR.G |  |
| R.TSVLAAANPK.E |  |
| [R.TVSATLR.Q](http://ms-searching2.rockefeller.edu/mascot/cgi/peptide_view.pl?file=../data/20110304/F015212.dat&query=345&hit=2&index=gi|74025442&px=1&section=5) |  |
| R.VVVTGIYR.A |  |
|  |  |
| **MCM5 (Tb927.11.5570)** | **2,4, and 5** |
| K.ANLTTMLNSR.T |  |
| K.AQTPIIQVTAR.Q |  |
| K.CRPNPYTLLPMECEYEDQQIIK.V |  |
| K.DGGASVQSVNQNFSSR.V |  |
| K.LAQHVINLHK.G |  |
| [K.LLPPLSR.R](http://ms-searching2.rockefeller.edu/mascot/cgi/peptide_view.pl?file=../data/20110304/F015212.dat&query=411&hit=1&index=gi|71755435&px=1&section=5) |  |
| K.LLPPLSRR.S |  |
| K.REELEWR.R |  |
| K.SATVDAIK.S |  |
| K.SGVSDQSMTAAQSELVLR.I |  |
| K.VQELPEDVPTGELPR.H |  |
| R.AAIQCTSCSSK.T |  |
| R.ADAEEAIR.L |  |
| R.AIYAMMK.R |  |
| R.ALPLFEHAVWELAQEHK.L |  |
| [R.CVGLMFITAK.D](http://ms-searching2.rockefeller.edu/mascot/cgi/peptide_view.pl?file=../data/20110304/F015212.dat&query=2059&hit=2&index=gi|71755435&px=1&section=5) |  |
| R.EAHQQTLDALSNSGGTK.A |  |
| R.EELEWR.R |  |
| R.EQDQVAIHEAMEQQTISIAK.A |  |
| R.EQDQVAIHEAMEQQTISIAK.A |  |
| R.FAEQGDVYER.L |  |
| R.GDMNVLFIGDPSTAK.S |  |
| R.GTATGGAGFGGGVGQR.K |  |
| R.HSHSTTTAVVER.S |  |
| R.HVTVVVDR.Y |  |
| R.IEDALR.R |  |
| R.IEDALRR.R |  |
| R.ISEDAMSVLLDFYVHVR.R |  |
| R.LAQSIDPAIFGLQDQK.K |  |
| [R.LAQSIDPAIFGLQDQKK.A](http://ms-searching2.rockefeller.edu/mascot/cgi/peptide_view.pl?file=../data/20110304/F015212.dat&query=5444&hit=1&index=gi|71755435&px=1&section=5) |  |
| [R.LDVLAHR.A](http://ms-searching2.rockefeller.edu/mascot/cgi/peptide_view.pl?file=../data/20110304/F015212.dat&query=545&hit=1&index=gi|71755435&px=1&section=5) |  |
| R.LMSEMAR.V |  |
| [R.LVCVSGIVVK.V](http://ms-searching2.rockefeller.edu/mascot/cgi/peptide_view.pl?file=../data/20110304/F015212.dat&query=1799&hit=1&index=gi|71755435&px=1&section=5) |  |
| R.MAWQVEEEAAFQR.F |  |
| R.QGSNYLR.G |  |
| R.QQDEAIDLK.L |  |
| R.SFFTK.Y |  |
| R.SNEDQMDFQSSILSR.F |  |
| [R.SNEDQMDFQSSILSR.F](http://ms-searching2.rockefeller.edu/mascot/cgi/peptide_view.pl?file=../data/20110304/F015212.dat&query=5015&hit=1&index=gi|71755435&px=1&section=5) |  |
| R.TQIHRLR.- |  |
| R.TSVLAAANPTLGSYDPLR.S |  |
| R.VALGATVEHSR.L |  |
| R.VALGATVEHSRLMSEMAR.V |  |
| R.VGFDVK.L |  |
| R.VHNDHLYMAMLR.G |  |
| R.YLVDR.V |  |
|  |  |
| **MCM6 (Tb927.11.11640)** | **4** |
| R.GVSGGVPGQAPQK.T |  |
| K.MTEAIKDSGDGAAER.E |  |
| K.DQVAIHEAMEQQTISIAK.A |  |
|  |  |
| **MCM7 (Tb927.11.16140)** | **2,4, and 5** |
| K.AAFAVAATSK.Y |  |
| [K.AGIITSLNAR.T](http://ms-searching2.rockefeller.edu/mascot/cgi/peptide_view.pl?file=../data/20110304/F015199.dat&query=1039&hit=2&index=gi|74026210&px=1&section=5) |  |
| K.DFLQAYVGEAK.K |  |
| K.EVVIEK.L |  |
| K.GGLIGTLVVLR.G |  |
| K.GIDESHLQR.C |  |
| [K.GSSGVGLTAAVTR.D](http://ms-searching2.rockefeller.edu/mascot/cgi/peptide_view.pl?file=../data/20110304/F015199.dat&query=1593&hit=1&index=gi|74026210&px=1&section=5) |  |
| K.IHPIVDSSAAK.V |  |
| K.IQVDNVK.Q |  |
| K.KIHPIVDSSAAK.V |  |
| [K.QHPDKEVVIEK.L](http://ms-searching2.rockefeller.edu/mascot/cgi/peptide_view.pl?file=../data/20110304/F015212.dat&query=3034&hit=3&index=gi|74026210&px=1&section=5) |  |
| K.VISDIYCEMR.A |  |
| K.VVGTYCPDPSTGQGHEAFR.A |  |
| K.WIASVAPR.S |  |
| K.YPNYLADR.D |  |
| R.DTYTGEVMLEGGALVLSDR.G |  |
| R.FFEEFR.D |  |
| R.GICCIDEFDK.M |  |
| R.GICCIDEFDKMDDSDR.T |  |
| R.GSIDLAEVRPALMIK.G |  |
| [R.HTNVVTAR.T](http://ms-searching2.rockefeller.edu/mascot/cgi/peptide_view.pl?file=../data/20110304/F015212.dat&query=917&hit=8&index=gi|74026210&px=1&section=5) |  |
| R.IATPGQVVK.V |  |
| R.IELER.R |  |
| R.IQMNVMGYR.E |  |
| [R.IQMNVMGYR.E](http://ms-searching2.rockefeller.edu/mascot/cgi/peptide_view.pl?file=../data/20110304/F015212.dat&query=2028&hit=1&index=gi|74026210&px=1&section=5) |  |
| R.IRSDTNICLMGDPGVAK.S |  |
| R.LLPQYK.A |  |
| R.LQELPQYVPR.G |  |
| R.MVTTSDAAIFSTIK.E |  |
| [R.MVTTSDAAIFSTIK.E](http://ms-searching2.rockefeller.edu/mascot/cgi/peptide_view.pl?file=../data/20110304/F015199.dat&query=3118&hit=1&index=gi|74026210&px=1&section=5) |  |
| R.NLTPSENVNLPPALLSR.F |  |
| R.RFFEEFR.D |  |
| R.RSYQEAADDLK.I |  |
| [R.SDTNICLMGDPGVAK.S](http://ms-searching2.rockefeller.edu/mascot/cgi/peptide_view.pl?file=../data/20110304/F015199.dat&query=3594&hit=2&index=gi|74026210&px=1&section=5) |  |
| R.SIFTTGK.G |  |
| R.SVAPEIWGMEDVK.K |  |
| R.SVAPEIWGMEDVKK.A |  |
| R.SYQEAADDLK.I |  |
| R.TALHEVMEQQMVSIAK.A |  |
| [R.TLLSIIR.L](http://ms-searching2.rockefeller.edu/mascot/cgi/peptide_view.pl?file=../data/20110304/F015212.dat&query=511&hit=5&index=gi|74026210&px=1&section=5) |  |
| R.TSILAAANPK.Y |  |
| R.VICEGEQTR.I |  |
| R.VVEADVR.E |  |
| K.IQVDNVK.Q |  |
|  |  |
| **MCM8 (Tb927.10.10410)** | **2,4, and 5** |
| K.AGMVFSVPVHTAILTAGNPIGGR.F |  |
| K.APTEDGVLVYPGR.C |  |
| K.EAIILAVVGGTAMK.K |  |
| K.SIPANLNLSPALFTR.F |  |
| K.TSGPPLPLETVQR.F |  |
| R.AACTVAPR.S |  |
| R.ACFVENSR.H |  |
| R.AEAAMGELAVTPR.F |  |
| R.FDIVICMR.S |  |
| R.FLQALIR.V |  |
| R.FLQALIRVSEAR.A |  |
| R.FYEMVR.N |  |
| R.GECEEVQLLK.V |  |
| R.GPMQTIDSLEESGR.F |  |
| R.GYNWAPLLER.G |  |
| [R.HEVTAEDAR.Y](http://ms-searching2.rockefeller.edu/mascot/cgi/peptide_view.pl?file=../data/20110304/F015210.dat&query=1208&hit=1&index=gi|71748414&px=1&section=5) |  |
| R.IDMMETPMR.N |  |
| R.LFAGFLAPLQIR.S |  |
| R.LVIPLLEFFK.A |  |
| R.NAVVGQPR.L |  |
| R.QIGELGSWR.L |  |
| R.RAEAAMGELAVTPR.F |  |
| R.SLSDHVLQLHR.C |  |
| R.TGVNALSHR.T |  |
| R.TNATQQIAVR.A |  |
| R.TVLAACEEAGCR.D |  |
| [R.YAVELMK.R](http://ms-searching2.rockefeller.edu/mascot/cgi/peptide_view.pl?file=../data/20110304/F015199.dat&query=497&hit=1&index=gi|71748414&px=1&section=5) |  |
|  |  |
| **Putative DNA topoisomerase II (Tb927.11.11540)** | **4** |
| R.LWNLTAEMSAR.L |  |
| K.ELILFSVADCER.S |  |
| K.DDDDIVMAFAKDK.V |  |
| R.FVNEVIDGTFIVTRR.S |  |
|  |  |
| **ALBA3 (Tb927.4.2040)** | **12** |
| K.ISAMGAAIR.S |  |
| K.GQGAENEIR.V |  |
| R.SAVGVAEVLKR.R |  |
| [K.EKGQGAENEIR.V](http://ms-searching2.rockefeller.edu/mascot/cgi/peptide_view.pl?file=../data/20110304/F015223.dat&query=2176&hit=1&index=gi|72387860&px=1&section=5) |  |
| K.EEEERDPVER.R |  |
|  |  |
| **Putative 40S ribosomal protein S4 (Tb927.11.3590)** | **10** |
| R.VSEAESSIK.M |  |
| R.HPGAFDIAR.L |  |
| K.VVMVTGGANR.G |  |
| R.LKDASGHEFATR.A |  |
|  |  |
| **Putative 40S ribosomal protein S3A (Tb927.10.3930)** | **10** |
| K.FTVQEVQGR.N |  |
| R.VVEEAQEETA. |  |
| K.TQNEDDAYR.K |  |
| K.LDINDAVSLLTR.N |  |
|  |  |
| **Putative 40S ribosomal protein SA (Tb927.11.10790)** | **10** |
| R.VLVVTDPR.T |  |
| R.FIPGTFTNQIQK.K |  |
| R.VIAAVENPQDVTVCSTR.L |  |
|  |  |
| **60S ribosomal protein L2 (Tb927.5.1110)** | **10** |
| K.AIEHEPGR.G |  |
| R.AMIGIVAGGGR.I |  |
|  |  |
| **Putative 60S ribosomal protein L5 (Tb927.9.15110)** | **10** |
| K.VSPDDMEGMYK.R |  |
| K.VSPDDMEGMYKR.A |  |
|  |  |
| **Putative 40S ribosomal protein S18 (Tb927.10.5330, Tb927.10.5340)** | **13** |
| R.QRDPK.T |  |
| K.KAGVDVER.R |  |
| K.TEHLTSSMVDTR.L |  |
|  |  |
| **Putative 60S acidic ribosomal subunit protein (Tb927.11.2050)** | **9** |
| K.VDNSTATLLQK.L |  |
| R.EDLSVTDAVVEK.Y |  |
| K.VLSTGDKVDNSTATLLQK.L |  |
| R.VGAIAPCDVIVPAGNTGMEPK.A |  |
|  |  |
| **Putative chaperone protein DNAj (Tb927.2.5160)** | 7 |
| K.LAIIRDR.L |  |
| K.VFDVVVEK.G |  |
| R.RYDQFGEK.G |  |
| R.EGMPIPGTGGTER.G |  |
| R.SIGPGFVQQMQVACPR.C |  |
|  |  |
| **Hypothetical conserved protein (Tb927.4.2640)** | **4** |
| K.QEEEALR.A |  |
| R.CGMAAATPARTSTPGR.S |  |
|  |  |
| **Putative RNA-binding protein (Tb927.6.3480)** | **2** |
| K.AVTLPSR.A |  |
| K.IMLDPSTGNSKGFGFVLFDK.E |  |
|  |  |
| **Putative myosin heavy chain (Tb927.11.16310)** | **4** |
| R.SPAELYK.R |  |
| R.GVVKAVPEGSGK.V |  |
| R.GYGVTETELASVR.R |  |
|  |  |
| **Putative 60S ribosomal protein L12 (Tb927.9.14000)** | **13** |
| K.ESAPNSMGASMK.S |  |
|  |  |
| **Hypothetical conserved protein (Tb927.4.3340)** | **5** |
| R.RAELSGHVVDGAMK.N |  |
|  |  |
| **Putative leucine-rich repeat protein (Tb927.6.1160)** | **6** |
| R.DGTATSMAAGKSDGK.E |  |
|  |  |
| **Glyceraldehyde 3-phosphate dehydrogenase, glycosomal (Tb927.6.4280)** | **9** |
| R.VVDLVR.H |  |
| [K.TVDGVSVK.D](http://ms-searching2.rockefeller.edu/mascot/cgi/peptide_view.pl?file=../data/20110304/F015217.dat&query=383&hit=4&index=gi|72390503&px=1&section=5) |  |
| K.EIDAALKR.A |  |
| R.YFAYQMK.Y |  |
| K.VVISAPASGGAK.T |  |
| [R.KVVISAPASGGAK.T](http://ms-searching2.rockefeller.edu/mascot/cgi/peptide_view.pl?file=../data/20110304/F015217.dat&query=1997&hit=1&index=gi|72390503&px=1&section=5) |  |
| K.AVGMVIPSTQGK.L |  |
| [K.ATLQNNLPNER.R](http://ms-searching2.rockefeller.edu/mascot/cgi/peptide_view.pl?file=../data/20110304/F015217.dat&query=2456&hit=1&index=gi|72390503&px=1&section=5) |  |
| R.AAALNIIPSTTGAAK.A |  |
| [K.TFVMGVNHNDYNPR.E](http://ms-searching2.rockefeller.edu/mascot/cgi/peptide_view.pl?file=../data/20110304/F015217.dat&query=4408&hit=1&index=gi|72390503&px=1&section=5) |  |
| R.VPTADVSVVDLTFIATR.D |  |
|  |  |
| **Hypothetical, conserved protein (Tb927.11.3510)** | **10** |
| R.SMFAEDDMQSR.Q |  |
| K.ASRPAHQVGTVEADNVNSAFDNTTAALGAPAVISDRR.E |  |
| K.EALLQWNPAVSANSDPVELHSTHVANYLQDASEMR.A |  |
