# Supplementary material for: MCM-BP Is Required for Repression of Life-Cycle Specific Genes Transcribed by RNA Polymerase I in the Mammalian Infectious Form of Trypanosoma brucei
Source: PLoS One. 2013 Feb 25;8(2):e57001. doi: 10.1371/journal.pone.0057001 (PMC3581582; doi:10.1371/journal.pone.0057001)
Supplement: Table S4 — Trypanosoma brucei strains used in this study. (DOC) [file pone.0057001.s006.doc]

**Supporting Table S4**. *Trypanosoma brucei* strains used in this study

| Names | Life-cycle | Genotypes | Sources |
| --- | --- | --- | --- |
| PF427 | PF | Wild type |  |
| HSTB-10 | PF | The BES11 promoter::*PUR-LUC-emGFP* (pHJ1) | This study |
| HSTB-39 | PF | The BES11 promoter::*PUR-LUC-emGFP* (pHJ1)  *Transposase::SAT* (pHJ2) | This study |
| HSTB-590 | PF | *mcm-BP∆HYG-TK / MCM-BP-PTP::NEO* | This study |
| SM | BF | T7 RNA polymerase and Tet repressor (TetR)::*NEO* | [1] |
| HSTB-188 | BF | The BES1 promoter::*BSD* | [2] |
| HSTB-317 | BF | The BES1 promoter::*BSD*, *mcm-BP∆PUR-TK / MCM-BP* | This study |
| HSTB-464, 465 | BF | The BES1 promoter::BSD, *mcm-BP∆PUR-TK / MCM-BP-myc::PHLEO* + *MCM-BP RNAi::HYG* (pHJ35) | This study |
| HSTB-537 | BF | *MCM-BP-myc::PHLEO* | This study |
| HSTB-572 | BF | *MCM-BP-myc::PHLEO*, *MCM2-flag::HYG* | This study |
| HSTB-600 | BF | *MCM-BP-myc::PHLEO*, *MCM5-flag::HYG* | This study |
| HSTB-570 | BF | *MCM2-flag::HYG* | This study |
| HSTB-594 | BF | *MCM5-flag::HYG* | This study |
| HSTB-660 | BF | The BES1 promoter::*BSD*, *mcm-BP∆ / loxP-MCM-BP-myc-HYG-TK-loxP* + pLew100-Cre-EP1 | This study |
| HSTB-683 | BF | The BES1 promoter::*BSD*,  A silent BES promoter::*PUR-LUC-emGFP* (pHJ1)  *mcm-BP∆ / loxP-MCM-BP-myc-HYG-TK-loxP* + pLew100-Cre-EP1 | This study |
| HSTB-684 | BF | The BES1 promoter::*BSD*,  A silent BES promoter::*PUR-LUC-emGFP* (pHJ1)  *mcm-BP∆ / loxP-MCM-BP-myc-HYG-TK-loxP* + pLew100-Cre-EP1 | This study |

**References**

1. Wirtz E, Leal S, Ochatt C, Cross GAM (1999) A tightly regulated inducible expression system for dominant negative approaches in *Trypanosoma brucei*. Mol Biochem Parasitol 99: 89-101.

2. Kim HS, Cross GA (2010) TOPO3alpha influences antigenic variation by monitoring expression-site-associated VSG switching in *Trypanosoma brucei*. PLoS Pathog 6: e1000992.
