# Supplementary material for: MCM-BP Is Required for Repression of Life-Cycle Specific Genes Transcribed by RNA Polymerase I in the Mammalian Infectious Form of Trypanosoma brucei
Source: PLoS One. 2013 Feb 25;8(2):e57001. doi: 10.1371/journal.pone.0057001 (PMC3581582; doi:10.1371/journal.pone.0057001)
Supplement: Table S5 — Plasmids used in this study. (DOC) [file pone.0057001.s007.doc]

**Supporting Table S5.** Plasmids used in this study

| Names | Inserts (markers) and targeting loci | Sources |
| --- | --- | --- |
| pHJ1 | Triple reporter with *PUR*-*LUC*-*EmGFP* targeting at a BES promoter | This study |
| pHJ2 | *Transposase* (*SAT*), *TUB* locus | This study |
| pSGL35 | Transposon donor (*NEO*) | [1] |
| pHJ17 | *loxP-HYG-TK-loxP* | [2] |
| pHJ18 | *loxP-PUR-TK-loxP* | [2] |
| pSY45 | *MCM-BP∆HYG-TK* at the original *MCM-BP* locus | This study |
| pHJ35 | *MCM-BP RNAi* (*HYG*), rDNA spacer | This study |
| pDS24 | *loxP-MCM-BP-myc-HYG-TK-loxP* at the original *MCM-BP* locus | This study |
| pDS15 (pMCM-BP-PTP-NEO) | C-terminal PTP tagging for *MCM-BP* (*NEO*) at the original *MCM-BP* locus | This study |
| pMOTag53M | One step PCR-3myc tagging (*PHELO*) | [3] |
| pMOTag4F | One step PCR-3myc tagging (*HYG*) | [3] |
| pLEW100-  Cre-EP1 | Cre-recombinase inducible vector (*PHLEO*), rDNA spacer | [4] |

**References**

1. Leal S, Acosta-Serrano A, Morris JC, Cross GAM (2004) Transposon mutagenesis of *Trypanosoma brucei* identifies glycosylation mutants resistant to Concanavalin A. J Biol Chem 279: 28979-28988.

2. Kim HS, Cross GA (2010) TOPO3alpha influences antigenic variation by monitoring expression-site-associated VSG switching in *Trypanosoma brucei*. PLoS Pathog 6: e1000992.

3. Oberholzer M, Morand S, Kunz S, Seebeck T (2006) A vector series for rapid PCR-mediated C-terminal in situ tagging of *Trypanosoma brucei* genes. Mol Biochem Parasitol 145: 117-120.

4. Scahill MD, Pastar I, Cross GA (2008) CRE recombinase-based positive-negative selection systems for genetic manipulation in *Trypanosoma brucei*. Mol Biochem Parasitol 157: 73-82.
